# Invariant Set Distributed Explicit Reference Governors for Provably Safe On-Board Control of Nano-Quadrotor Swarms

**DOI:** 10.3389/frobt.2021.663809

**Published:** 2021-06-22

**Authors:** Bryan Convens, Kelly Merckaert, Bram Vanderborght, Marco M. Nicotra

**Affiliations:** ^1^ Robotics and Multibody Mechanics (R&MM), Department of Mechanical Engineering, Vrije Universiteit Brussel, Brussels, Belgium; ^2^ Imec, Leuven, Belgium; ^3^ Flanders Make, Leuven, Belgium; ^4^ Robotics, Optimization, and Constrained Control (ROCC), Department of Electrical, Computer, and Energy Engineering, University of Colorado Boulder, Boulder, CO, United States

**Keywords:** aerial robotics control, multi-robot systems, actuator saturation, distributed collision avoidance, guaranteed safety, human-swarm interaction, invariant set control, nano-quadrotor swarm

## Abstract

This article provides a theory for provably safe and computationally efficient distributed constrained control, and describes an application to a swarm of nano-quadrotors with limited on-board hardware and subject to multiple state and input constraints. We provide a formal extension of the explicit reference governor framework to address the case of distributed systems. The efficacy, robustness, and scalability of the proposed theory is demonstrated by an extensive experimental validation campaign and a comparative simulation study on single and multiple nano-quadrotors. The control strategy is implemented in real-time on-board palm-sized unmanned erial vehicles, and achieves safe swarm coordination without relying on any offline trajectory computations.

## 1 Introduction

Swarms of aerial robots or Unmanned Aerial Vehicles (UAVs) are emerging as a disruptive technology that enables highly re-configurable, on-demand, distributed intelligent autonomous systems with high impact on many areas of science, technology, and society (Chung et al., 2018).

These swarms can be employed to solve real-world tasks where the environment is to be explored (Marconi et al., 2012; Bayram et al., 2017), and to be traversed or exploited (Vásárhelyi et al., 2018) with a prescribed goal state or a desired formation. To operate effectively in uncertain real-world environments, each agent in the swarm must be capable of safely navigating to its target along a-priori unknown paths. Not only does each robot need to respect its operational constraints (e.g. actuator saturation, speed limits, allowed flight zones), it must also avoid collisions with environmental hazards and other agents (Franchi et al., 2012; Alonso-Mora et al., 2015; Franchi et al., 2016; Zhou et al., 2018) in the presence of imperfect dynamic models, measurement noise, and communication delays. Most importantly, to ensure a high level of safety and robustness, the robots should use their on-board computational resources rather than relying on off-board resources (e.g. a ground control station). The latter provide a central point of failure, and are susceptible to time delays, communication overhead, and information loss. This calls for reactive and distributed control algorithms that can be implemented in real-time on-board UAVs and only rely on local information to solve the global navigation task safely.

Achieving goal satisfaction and safety certificates for a swarm of autonomous Micro Aerial Vehicles (MAVs) presenting limited resources for on-board computation, power, communication, sensing, and actuation is considerably challenging (Chung et al., 2018). Moreover, even for large platforms with more advanced capabilities, the computational power available to implement control algorithms is typically limited in favor of running mission-dependent algorithms related to localization and sensing systems (Brockers et al., 2014). Hence, computationally efficient and provably safe on-board algorithms for multi-robot systems are of paramount importance for achieving safety-critical tasks in complex environments.

In this work, we develop a provably safe and robust constrained control methodology that is fully distributed and can be implemented on the individual agents of a swarm of Vertical Take-Off and Landing (VTOL) vehicles. The algorithm is validated using the smallest open-source available nano-quadrotor platform, i.e. Bitcraze’s Crazyflie 2.1. An accompanying video can be found at https://youtu.be/le6WSeyTXNU.

## 2 Related Work

As discussed in (Murray, 2007; Brambilla et al., 2013; Parker et al., 2016; Chamanbaz et al., 2017; Chung et al., 2018; Coppola et al., 2020), swarm robotics has become an active area of research covering a broad spectrum of topics within the robotics and control communities. The problem of safely controlling the motion of aerial robot swarms can be classified based on approaches for which the main portion of the algorithm, and especially the part that ensures safety and goal satisfaction, is running either *off-board* or *on-board* the UAVs. This classification is motivated because most existing works provide algorithmic contributions which belong to the off-board category (see Section 2.1), but as explained in Section 1, on-board navigation algorithms (see Section 2.2) are preferred from a safety and autonomy perspective.

Unfortunately, there does not exist one safe navigation strategy that suits all UAV applications. For each strategy there is an inherent trade-off between computational efficiency, performance, safety guarantees, simplicity, generality, and scalability to swarms. To provide a fair point of comparison, it is worth noting that VTOLs can vary significantly in terms of the available on-board computational power. For instance, a 35 g Crazyflie quadrotor carries an STM32F4 microprocessor with a clock speed of 168MHz and 192kB RAM. For comparison, larger platforms with a mass above ±700 g can use processors like the Odroid-XU4 (Liu et al., 2018) or the NVIDIA TX2 (Jung et al., 2018; Sanket et al., 2018; Ding et al., 2019; Carrio et al., 2020). The latter has a six-core CPU, each with a clock speed of 2GHz, a 256-core NVIDIA GPU, and 8 GB RAM. Since very limited battery power for computation, memory, and communication available to tiny MAVs intrinsically calls for different kinds of navigation and control strategies (Purohit et al., 2014), the literature review is mainly limited to off-board and on-board navigation strategies applied to nano-quadrotors.

### 2.1 Off-Board Navigation Strategies for Nano-Quadrotors

Most approaches, such as (Campos-Macías et al., 2017; Chen et al., 2017; Herbert et al., 2017; Preiss et al., 2017a; Wang et al., 2017; Fridovich-Keil et al., 2018; Honig et al., 2018; Kolaric et al., 2018; Cappo et al., 2018a; Cappo et al., 2018b; Xu and Sreenath, 2018; Bajcsy et al., 2019; Du et al., 2019; Fathian et al., 2019; Liu et al., 2019; Luis and Schoellig, 2019; Rubies-Royo et al., 2019; Vukosavljev et al., 2019), try to ensure a particular level of safety and robustness, by running the core search-based or optimization-based algorithms *off-board* the UAVs, and thus outsource the high computational cost to ground control stations that send the trajectories to the UAV’s on-board position or attitude controller. Frameworks such as (Preiss et al., 2017a; Honig et al., 2018) combine graph-based planning and continuous trajectory optimization to compute safe and smooth trajectories, but take several minutes for a swarm of hundreds of quadrotors in obstacle-rich environments. In (Luis and Schoellig, 2019), a scalable distributed model predictive control algorithm with on-demand collision avoidance is proposed to perform point-to-point transitions with labeled agents. This strategy reduces the computation time to the order of seconds. (Campos-Macías et al., 2017) introduces a hybrid approach to trajectory planning, fusing sampling-based planning techniques and model-based optimization via quadratic programming (QP). For a single nano-quadrotor in obstacle-dense environments, a provably safe trajectory can be computed online every 0.1–1s, depending on the scenario. Frameworks such as (Du et al., 2019; Vukosavljev et al., 2019) are based on designing off-board libraries of safe motion primitives for a swarm of tiny MAVs, but typically require too much memory for on-board implementation. (Du et al., 2019) relies on combinatorial and nonlinear optimization techniques that are executed on a central computer, requires iterative procedures to resolve collisions between agents in a sequential manner, and does not guarantee to find a feasible solution. A modular, robust, and hierarchical framework for safe planning of robot teams is proposed in (Vukosavljev et al., 2019). Although the run-time components, executed off-board, require only a small computing time, this approach is centralized, requires a-priori known environments and is conservative due to the restriction to a discretization, i.e. a gridded workspace partitioned into rectangular boxes. Works based on the online FaSTrack motion planner (Herbert et al., 2017) provide strong safety guarantees under the assumption of a single near-hover quadrotor with a decoupled structure (Fridovich-Keil et al., 2018) or obtain weaker safety guarantees using neural network classifiers to consider control-affine dynamics (Rubies-Royo et al., 2019). Hamilton-Jacobi reachability analysis was applied to multi-agent swarms using sequential priority ordering (Bajcsy et al., 2019) or the selection of air highways (Chen et al., 2017). A centralized multi-robot system planner for enabling theatrical performance is designed in (Cappo et al., 2018a; Cappo et al., 2018b) using time-aware trajectory formulation for validation, verification, and trajectory refinement. The human intent is translated online into non-colliding and dynamically feasible trajectories for multiple nano-quadrotors. Safety barrier certificates based on exponential control barrier functions are used in (Wang et al., 2017) to ensure in a minimally invasive way collision-free maneuvers for teams of small quadrotors flying through formations and in (Xu and Sreenath, 2018) for the safe teleoperation of nano-quadrotor swarms via a remote joystick in a set of static constraints. In (Wang et al., 2017) this requires a centralized QP to be solved at 50 Hz on a ground PC to minimize the difference between the actual and nominal control. Distributed formation control approaches that have been demonstrated on small quadrotors, but are computed off-board have shown robustness to bounded measurement noise (Kolaric et al., 2018), to communication delays, nonlinearities, parametric perturbations, and external disturbances (Liu et al., 2019). Input feasibility and collision avoidance is guaranteed in (Fathian et al., 2019) for single-integrator dynamics, and is claimed to be extendable to agents with higher-order dynamics in (Fathian et al., 2018).

### 2.2 On-Board Navigation Strategies for Nano-Quadrotors

Only few works such as (Preiss et al., 2017b; Desaraju and Michael, 2018; McGuire et al., 2019) achieved to run computationally efficient navigation algorithms *on-board* the small embedded flight controllers of nano-quadrotors, but mostly with limited safety guarantees. These strategies typically can only handle first order dynamics, can only deal with a small set of constraints and a small number of agents, or require too much on-board memory. In (McGuire et al., 2019), a swarm gradient bug algorithm reacts to static obstacles on the fly, but collisions still occur. In (Preiss et al., 2017b), single piece polynomial planners can follow predefined paths uploaded offline for a single quadrotor, but are not suitable for dynamically changing environments. They use artificial potential fields on a swarm of these UAVs hovering in formation and show avoidance of an obstacle with a known position in a distributed fashion, but without providing theoretical safety certificates on collision avoidance or actuator saturation. A promising approach to the computationally efficient robust constrained control of nonlinear systems is proposed in (Desaraju et al., 2018) and uses an experience driven Explicit MPC (EMPC). This method was implemented in (Desaraju and Michael, 2018) and reliably ran at 100 Hz on board the tiny MAV’s firmware in the presence of control input and velocity constraints. Due to the nature of EMPC, however, the introduction of collision avoidance constraints between multiple robots would make the EMPC database grow exponentially in size, thus becoming prohibitive for fast online queries.

### 2.3 Contributions

To the best of our knowledge, the literature does not provide any provably safe control techniques that achieve on-board real-time control of large nano-quadrotor swarms with higher-order dynamics in the presence of actuator, obstacle, and agent collision avoidance constraints.

This work is based on the Explicit Reference Governor (ERG), which is a novel framework for the closed-form feedback control of nonlinear systems subject to constraints on the state and input variables (Nicotra and Garone, 2018). This approach does not rely on online optimization and is particularly promising for control applications with fast dynamics, limited on-board computational capabilities, or strict regulations on code reliability. This article extends the centralized ERG framework (Nicotra and Garone, 2018) and a distributed ERG (D-ERG) (Nicotra et al., 2015) formulation, and encapsulates these two core contributions:

1. The ERG theory is extended to distributed multi-agent systems with fourth-order dynamics and subject to constraints on states and actuator inputs. This work supplies all theoretical details of a general and scalable D-ERG framework along with a formal proof on correctness, the formulation of different offline design strategies for computing safe threshold values of Lyapunov and invariance-based level sets. Moreover we formulated two swarm collision avoidance control policies, a decentralized and a distributed version, that require a different information exchange.
2. The effectiveness, robustness, and computational efficiency of our control and navigation layers, running on-board the Crazyflie nano-quadrotor at 500 Hz, is validated extensively in several scenarios with single or multiple quadrotors subject to state and input constraints. All proposed formulations are validated and quantitatively compared. These are the first published experimental results on the use of ERG and D-ERG on quadrotors, and (to the best of our knowledge) is the only work in the literature that achieves provably safe constrained control at such high frequencies on-board nano-quadrotors for such a broad set of state and input constraints. The D-ERG’s goal satisfaction and safety certificates are put in sharp contrast with those of a Navigation Field method that suffers from instabilities and collisions when the agents posses higher-order dynamics.

The rest of this article is organized as follows. Section 3 introduces the used notation. The problem is formulated in Section 4. The proposed strategy is outlined in Section 5, and constitutes the control layer and the navigation layer which are described in Section 6 and in Section 7, respectively. The results of extensive hardware validations and a comparative simulation study with single and swarms of nano-quadrotors are presented in Section 8, and discussed in Section 9. Finally, some concluding remarks are given in Section 10.

## 3 Notation

In this work, all vectors are column vectors. Unit vectors are denoted using the hat symbol $\hat{a}$. Unit vectors aligned with the axes of a right-handed Cartesian reference frame are denoted as ${\hat{e}}_{1}$, ${\hat{e}}_{2}$, ${\hat{e}}_{3}$. $0_{m\times n}$ and $1_{m\times n}$ represent m×n matrices of zeros and ones, respectively. $I_{n}$ represents an identity matrix of dimension n×n. The concatenation of vectors $v_{i}$ to $v_{k}$ is denoted by the vector $v_{i:k}={\left[v_{i}^{T},\ldots ,v_{k}^{T}\right]}^{T}$. Given a vector in ${\mathbb{R}}^{3}$, ${\left\|\right\|}_{xy}$ denotes the following norm ${\left\|v\right\|}_{xy}=\sqrt{v_{1}^{2}+v_{2}^{2}}$. The hat operator $\wedge :{\mathbb{R}}^{3}\mapsto \text{SO}\left(3\right)$ denotes the skew-symmetric matrix transformation

$$v^{\wedge }=\left[\begin{array}{ccc} 0 & -v_{3} & v_{2}\\ v_{3} & 0 & -v_{1}\\ -v_{2} & v_{1} & 0 \end{array}\right],$$ (1)

whereas the vee operator $\vee :\text{SO}\left(3\right)\mapsto {\mathbb{R}}^{3}$ denotes the vector extraction of the skew-symmetric terms

$$R^{\vee }=\frac{1}{2}\left[\begin{array}{c} R_{32}-R_{23}\\ R_{13}-R_{31}\\ R_{21}-R_{12} \end{array}\right].$$ (2)

## 4 Problem Formulation

The system and parts of the problem are stated first. Section 4.1 presents the dynamic model of a generic quadrotor. Nevertheless, the proposed method can be readily extended to any VTOL vehicle. The state and input constraints, which each agent should always satisfy, are defined in Section 4.2 and illustrated in this video https://youtu.be/le6WSeyTXNU.

### 4.1 Dynamic Model

**FIGURE 1:**
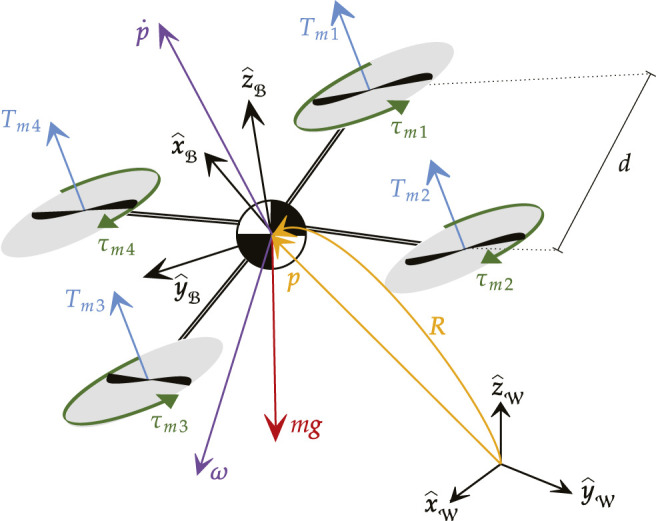
Schematic representation of a quadrotor agent.

As depicted in Figure 1, each agent of the robotic swarm is modeled as a quadrotor with mass $m\in {\mathbb{R}}_{>0}$ and moment of inertia $J\in {\mathbb{R}}_{>0}^{3\times 3}$, $J=J^{T}$ defined with respect to the body reference frame ℬ. Let $p={\left[x,y,z\right]}^{T}\in {\mathbb{R}}^{3}$ and $\dot{p}={\left[\dot{x},\dot{y},\dot{z}\right]}^{T}\in {\mathbb{R}}^{3}$ denote the position and the velocity of the body reference frame ℬ with respect to the inertial reference frame W. The attitude of each agent is represented by either the rotation matrix R or by the roll, pitch, and yaw angles $\Theta ={\left[\phi ,\theta ,\psi \right]}^{T}\in {\mathbb{R}}^{3}$ that realign the axes of ℬ with the axes of W. Finally, $\omega =\omega_{x}{\hat{x}}_{ℬ}+\omega_{y}{\hat{y}}_{ℬ}+\omega_{z}{\hat{z}}_{ℬ}\in {\mathbb{R}}^{3}$ denotes the angular velocity of the vehicle expressed in the frame ℬ.

As detailed in (Hua et al., 2013), the dynamic model of a generic VTOL subject to a gravitational force in the $-{\hat{z}}_{W}$ direction, a unidrectional thrust force $T\in {\mathbb{R}}_{\geq 0}$ in the ${\hat{z}}_{ℬ}$ direction, and a torque vector $\tau \in {\mathbb{R}}^{3}$ about the axes of ℬ is

$$\{\begin{array}{ll} \text{Position}\;\text{dynamics}: & m\ddot{p}=T{\hat{z}}_{ℬ}+mg,\\ \text{Attitude}\;\text{dynamics}: & J\dot{\omega }=-\omega^{\wedge }J\omega +\tau ,\\ & \dot{R}=\omega^{\wedge }R, \end{array}$$ (3)

where ${\hat{z}}_{ℬ}=R{\hat{e}}_{3}$, $g=-g{\hat{e}}_{3}$, and $g\approx 9.81\;\text{m}/{\text{s}}^{2}$ is the gravitational acceleration. System (3) possesses fourth-order dynamics and can be entirely described by the state vector

$$x={\left[p^{T},\Theta^{T},{\dot{p}}^{T},\omega^{T}\right]}^{T}\in {\mathbb{R}}^{12}$$ (4)

subject to the control input vector

$$u={\left[T,\tau^{T}\right]}^{T}\in {\mathbb{R}}^{4}.$$ (5)

For the specific case of a quadrotor, it is possible to rewrite the control input (5) as a function of the motor voltage commands $U={\left[U_{1},\ldots ,U_{4}\right]}^{T}\in {\mathbb{R}}^{4}$, leading to

$$u=\left[\begin{array}{cccc} K_{T} & K_{T} & K_{T} & K_{T}\\ -K_{T}\frac{d}{\sqrt{2}} & -K_{T}\frac{d}{\sqrt{2}} & K_{T}\frac{d}{\sqrt{2}} & K_{T}\frac{d}{\sqrt{2}}\\ -K_{T}\frac{d}{\sqrt{2}} & K_{T}\frac{d}{\sqrt{2}} & K_{T}\frac{d}{\sqrt{2}} & -K_{T}\frac{d}{\sqrt{2}}\\ -K_{\tau } & K_{\tau } & -K_{\tau } & K_{\tau } \end{array}\right]\left[\begin{array}{c} U_{1}^{2}\\ U_{2}^{2}\\ U_{3}^{2}\\ U_{4}^{2} \end{array}\right]$$ (6)

where *d* is the nominal distance between the motor axis and the center of mass of the aircraft, and $K_{T},K_{\tau }\in {\mathbb{R}}_{>0}$ denote the actuator’s thrust and torque constant respectively.

### 4.2 State and Input Constraints

To ensure safety of a swarm of $N_{\text{a}}$ agents, every agent $i\in \left\{1,\ldots ,N_{\text{a}}\right\}$ is subject to the following constraints.

#### 4.2.1 Saturation (Static Box Input Constraints)

Actuator saturation has been observed as the primary cause of instability for quadrotors in free flight. Indeed, whenever one of the motors is subject to saturation, the control law is unable to generate an arbitrary torque vector. This can lead to undesired attitude oscillations that quickly devolve into catastrophic failures. To prevent this scenario, each motor voltage $U_{j}$ is required to stay within its lower and upper saturation limits,

$$U_{\min}\leq U_{j}\leq U_{\max},\;\forall j\in \left\{1,2,3,4\right\},$$ (7)

with $U_{\min}<U_{h}=\sqrt{\mathrm{mg}/\left(4K_{T}\right)}<U_{\max}\in {\mathbb{R}}_{>0}$ and $U_{h}$ defines the motor voltages required for static hovering in place.

#### 4.2.2 Walls (Static Polytopic State Constraints)

All agents have collision radius $R_{\text{a}}\in {\mathbb{R}}_{>0}$ and are required to operate in a confined environment defined by a convex polytope of $N_{\text{w}}$ oriented faces (i.e. planar walls). To enforce this requirement, each agent *i* must satisfy the following convex constraint

$${\hat{c}}_{w_{j}}^{T}p_{i}\leq d_{w_{j}}-R_{a},\;\forall j\in \left\{1,\ldots ,N_{w}\right\},$$ (8)

with ${\hat{c}}_{w_{j}}\in {\mathbb{R}}^{3}$ denoting the normal vector on the wall pointing in the inadmissible direction and $d_{{\text{w}}_{j}}\in \mathbb{R}$ describing the shortest distance between the origin of W and the wall.

#### 4.2.3 Obstacles (Static Cylindrical/Spherical State Constraints)

In addition to planar walls, all agents must also avoid collision with $N_{o}$ cylindrical obstacles. To enforce this requirement, each agent *i* must satisfy the following non-convex constraints

$${\left\|p_{i}-o_{j}\right\|}_{xy}\geq R_{o_{j}}+R_{a},\;\forall j\in \left\{1,\ldots ,N_{o}\right\},$$ (9)

with cylinder radius $R_{o_{j}}\in {\mathbb{R}}_{>0}$ and center $o_{j}\in {\mathbb{R}}^{3}$. Note that the cylindrical obstacles can be replaced with spheres by replacing ${\left\|\right\|}_{xy}$ with the Eucledian norm.

#### 4.2.4 Agent Collisions (Collaborative Cylindrical/Spherical State Constraints)

To prevent undesirable interactions between agents (e.g. collision, propeller downwash, sonar jamming), each pair of agents is tasked with satisfying the following dynamic cylindrical exclusion constraints

$${\left\|p_{i}-p_{k}\right\|}_{xy}\geq 2R_{a},\;\forall k\in \left\{1,\ldots ,N_{a}\right\}:k\neq i.$$ (10)

As per the previous case, it is trivial to replace the cylindrical constraint with a spherical constraint if vertical agent interactions are not deemed problematic.

### 4.3 Control Objectives

The aim of this paper is to develop a guaranteed safe distributed constrained control strategy for an homogeneous swarm of quadrotors with very limited on-board resources for computation, memory, and communication. It is assumed that all agents are collaborative and that the locations of all nearby obstacles are known within the MAV’s limited sensing range. Let each agent be subject to an a priori unknown and arbitrary reference $r_{i}\left(t\right)={\left[p_{i}^{r}{\left(t\right)}^{T},\psi_{i}^{r}\left(t\right)\right]}^{T}\in {\mathbb{R}}^{4}$, where $p_{i}^{r}$ and $\psi_{i}^{r}$ are the target position and yaw of agent *i*. The aggregate reference for the swarm, denoted by $r_{1:N_{a}}\left(t\right)$, is steady-state admissible at time *t* if $p_{1:N_{a}}^{r}\left(t\right)$ satisfies constraints (8)–(10).

The purpose of this paper is to design a feedback control law in the form $U_{1:N_{a}}\left(r_{1:N_{a}}\left(t\right),x_{1:N_{a}}\left(t\right)\right)$ such that the following objectives are achieved for a suitably large set of initial conditions $x_{1:N_{a}}\left(0\right)$:

• Safety: For any piecewise continuous reference $r_{1:N_{a}}\left(t\right)$, the control law is able to guarantee constraint satisfaction, i.e. the set of constraints (7)–(10) on the state and input variables of all agents $c\left(x_{1:N_{a}}\left(t\right),U_{1:N_{a}}\left(t\right)\right)\geq 0,\forall t\geq 0$;
• Asymptotic Stability: If the reference $r_{1:N_{\text{a}}}$ is constant and steady-state admissible, the closed-loop system satisfies $\lim_{t\to \infty }\left({\left[p_{1:N_{a}}{\left(t\right)}^{T},\psi_{1:N_{a}}\left(t\right)\right]}^{T}\right)=r_{1:N_{a}}$;
• Robustness: The control law must ensure safety and stability in the presence of model uncertainty, sensor noise, and external disturbances;
• Reactiveness: The control law must run in real-time on-board the nano-quadrotor’s hardware, without relying on off-board pre-generated trajectories;
• Scalability: Each agent must be capable of generating its own control input based on local information. To this end, inter-agent communication is limited to a given radius.

## 5 Proposed Strategy

The main challenge that arises from the control problem stated in Section 4.3 is that it combines the nonlinear dynamics of the individual agent with the nonconvex constraints of the aggregated swarm. The higher-order nonlinear agent dynamics (3) would be significantly easier to stabilize in the absence of constraints, whereas the position constraints (8)–(10) would be easier to enforce if the agent dynamics were a first-order linear system ${\dot{p}}_{i}=\rho_{i}$ as in (Fathian et al., 2019). We propose a multi-layer control architecture that relies on the ERG framework (Nicotra and Garone, 2018) and decouples the control problem into more tractable sub-tasks to facilitate on-board implementation.

**FIGURE 2:**
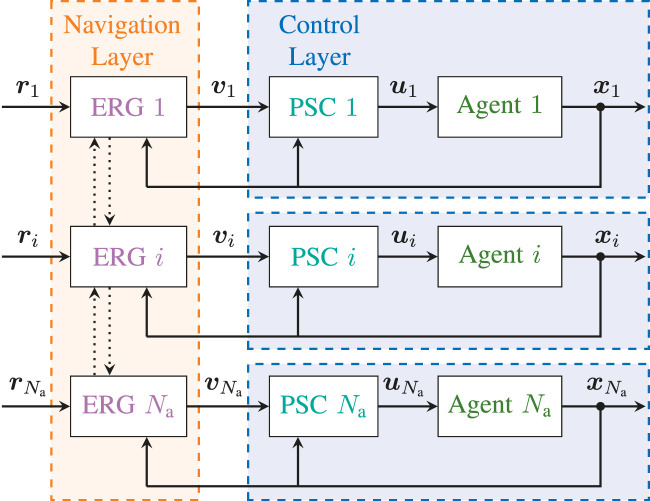
Distributed Constrained Control Architecture − The higher-order dynamics of each agent in the multi-robot system are stabilized by a Pre-Stabilizing Control (PSC) unit that computes the control inputs $u_{i}$ using only $x_{i}$ for state feedback and without accounting for constraints. An Explicit Reference Governor (ERG) block is placed in a distributed fashion before each pre-stabilized agent and only relies on information $v_{N_{i}}$ available in its local one-hop spherical neighborhood $N_{i}$ to enforce state and input constraints and achieve asymptotic convergence to $r_{i}$. In this article $v_{N_{i}}$ represents the set of applied references $v_{k}$ in the distributed policy or the set of states $x_{k}$ in the decentralized policy (such that a worst-case approximation of $v_{k}$ can be locally computed) for all agents *k* in the one-hop local neighborhood of agent *i*. We assume each agent can communicate in parallel with its neighbors.

The first task, which is handled by the **Control Layer**, consists in pre-stabilizing the dynamics of each agent to a locally defined reference $v_{i}\left(t\right)={\left[p_{i}^{v}{\left(t\right)}^{T},\psi_{i}^{v}\left(t\right)\right]}^{T}\in {\mathbb{R}}^{4}$. This will be done using a classical inner-outer loop controller that does not account for system constraints and does not require any form of inter-agent coordination. The second task, which is handled by the **Navigation Layer**, consists in manipulating the aggregate auxiliary references $v_{1:N_{a}}\left(t\right)$ so that the constraints are always satisfied. This layer is also responsible for coordinating the overall swarm and reaching the target configuration $r_{1:N_{a}}\left(t\right)$. The proposed control architecture is illustrated in Figure 2. The detailed design of the control and navigation layers will be addressed in Sections 6 and 7, respectively.

## 6 Control Layer

**FIGURE 3:**
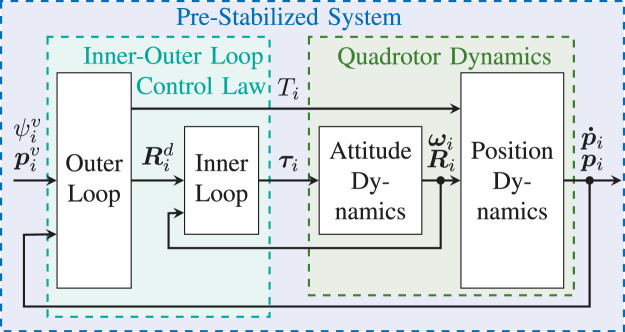
Pre-Stabilizing Control Scheme − In the traditional inner-outer loop control paradigm, it is assumed that the inner loop control law stabilizes the attitude dynamics an order of magnitude faster than the outer loop control law stabilizes the position dynamics.

The goal of the control layer is to pre-stabilize the individual quadrotors using a classical nonlinear inner-outer loop control law (Mellinger and Kumar, 2011; Hua et al., 2013). This is done without accounting for the state or input constraints, which will instead be handled by the navigation layer. The proposed architecture of the control layer is illustrated in Figure 3.

### 6.1 Inner-Outer Loop Control Law

The objective of the outer loop is to control the position of the quadrotor under the assumption that the attitude dynamics are instantaneous. To this end, we define the auxiliary control input $R^{d}\in \text{SO}\left(3\right)$ and assume that $R\approx R^{d}$. The position dynamics in the dynamic model (3) then become

$$m\ddot{p}=TR^{d}{\hat{e}}_{3}-mg{\hat{e}}_{3},$$ (11)

where $TR^{d}{\hat{e}}_{3}$ is the desired thrust vector expressed in W. Using a PD control law with gravity compensation, the outer loop control inputs *T* and $R^{d}$ are chosen so that

$$T^{d}=TR^{d}{\hat{e}}_{3}=m\left(K_{P}\left(p^{v}-p\right)-K_{D}\dot{p}+g{\hat{e}}_{3}\right),$$ (12)

where $K_{P}$, $K_{D}>0$ are diagonal gain matrices. The total thrust can thus be obtained as

$$u_{1}=T=m\left\|K_{P}\left(p^{v}-p\right)-K_{D}\dot{p}+g{\hat{e}}_{3}\right\|.$$ (13)

The target attitude is $R^{d}=R_{\psi^{v}}R_{\alpha^{d}}$, where $R_{\psi^{v}}$ is a standard rotation of $\psi^{v}$ around the third axis, whereas $R_{\alpha^{d}}$is the minimum rotation $\alpha^{d}$ that aligns ${\hat{z}}_{W}$ with the desired ${\hat{z}}_{ℬ}^{d}=T^{d}/T$ and one can obtain it using the Rodrigues formula with $\alpha^{d}=\arctan\left(\sqrt{{\left(T_{x}^{d}\right)}^{2}+{\left(T_{y}^{d}\right)}^{2}}/T_{z}^{d}\right)$.

The objective of the inner loop is to control the attitude dynamics of the UAV such that the rotation matrix R asymptotically tends to a constant $R^{d}$. As detailed in Lee (2011), a possible strategy to compute the torque vector is to define the attitude error as

$$e_{R}=\frac{1}{2}{\left(R^{dT}R-R^{T}R^{d}\right)}^{\vee },$$ (14)

and compute the control torques as follows,

$$\tau =-K_{R}e_{R}-K_{\omega }\omega ,$$ (15)

where $K_{R}$, $K_{\omega }>0$ are diagonal gain matrices.

### 6.2 Robust Closed Loop Dynamics

The following Lemma states the robustness of the outer loop dynamics to attitude errors.

Lemma 1. Let system (3) be subject to the outer loop controller (12), with $K_{P},K_{D}>0$, and the inner loop controller (15), with $K_{R},K_{\omega }>0$. Assume that the inner loop dynamics are sufficiently fast with respect to the outer loop dynamics. Given a constant applied position reference $p^{v}$ and a constant applied yaw reference $\psi^{v}$, then

$$V\left(p,\dot{p},p^{v}\right)={\left[\begin{array}{c} p-p^{v}\\ \dot{p} \end{array}\right]}^{T}P\left[\begin{array}{c} p-p^{v}\\ \dot{p} \end{array}\right],$$ (16)

with

$$P=\frac{1}{2}\left[\begin{array}{cc} K_{P}+\varepsilon K_{D}^{2} & \varepsilon K_{D}\\ \varepsilon K_{D} & I_{3} \end{array}\right],$$ (17)

is a Lyapunov function of the outer loop dynamics ∀ε∈(0,1). Moreover, the outer loop is Input-to-State Stable (ISS) with restrictions on the attitude error.

*Proof*: Given ∀ε∈(0,1), (16) is an ISS-Lyapunov candidate function for the outer loop dynamics. Noting that for a non-ideal inner loop $R{\hat{e}}_{3}=RR^{d^{T}}R^{d}{\hat{e}}_{3}$, the closed loop position dynamics, obtained by combining (3) and (12), without assuming $R^{d}\approx R$, have the form

$$\ddot{p}=\tilde{R}K_{P}\left(p^{v}-p\right)-\tilde{R}K_{D}\dot{p}+\left(\tilde{R}-I_{3}\right)g{\hat{e}}_{3},$$ (18)

where $\tilde{R}=RR^{d^{T}}$ represents the attitude error. Equation (18) is a Linear Parameter Varying (LPV) system that can be written in state-space form

$$\left[\begin{array}{c} \dot{p}\\ \ddot{p} \end{array}\right]=f\left(p,\dot{p},p^{v}\right)=A\left(\tilde{R}\right)\left[\begin{array}{c} p\\ \dot{p} \end{array}\right]+B\left(\tilde{R}\right)\left[\begin{array}{c} p^{v}\\ 0_{3\times 1} \end{array}\right]+d\left(\tilde{R}\right),$$ (19)

with

$$\begin{array}{l} A\left(\tilde{R}\right)=\left[\begin{array}{cc} 0_{3\times 3} & I_{3}\\ -\tilde{R}K_{P} & -\tilde{R}K_{D} \end{array}\right],\\ B\left(\tilde{R}\right)=\left[\begin{array}{cc} 0_{3\times 3} & 0_{3\times 3}\\ \tilde{R}K_{P} & 0_{3\times 3} \end{array}\right]. \end{array}$$

Noting that $A{\left(I_{3}\right)}^{T}P+PA\left(I_{3}\right)<0$ as detailed in (Khalil, 2001, Example 4.5, pp. 121–122), it follows that $A{\left(\tilde{R}\right)}^{T}P+PA\left(\tilde{R}\right)\leq 0$ for $\tilde{R}$ sufficiently close to $I_{3}$ (i.e. for a sufficiently small attitude error). This shows that (18) is Input to State Stable (ISS) with respect to sufficiently small attitude errors.▪

## 7 Navigation Layer

### 7.1 Distributed Explicit Reference Governor

The ERG is a general framework for the constrained control of nonlinear systems introduced in (Garone and Nicotra, 2016; Nicotra and Garone, 2018). Consider a pre-stabilized system $\dot{x}=f\left(x,v\right)$ such that, if the applied reference v remains constant, the closed-loop equilibrium point ${\bar{x}}_{v}$ is asymptotically stable. Given a continuous steady-state admissible path $\Phi :\left[0,1\right]\to {\mathbb{R}}^{3}$ between an initial reference Φ(0)=v(0) and a target reference Φ(1)=r, the principle behind the ERG is to generate a reference v(t)∈{Φ(s)|s∈[0,1]} such that

- the transient dynamics of the closed-loop system cannot cause a constraint violation;
- $\lim_{t\to \infty }v\left(t\right)=\Phi \left(1\right)$.

**FIGURE 4:**
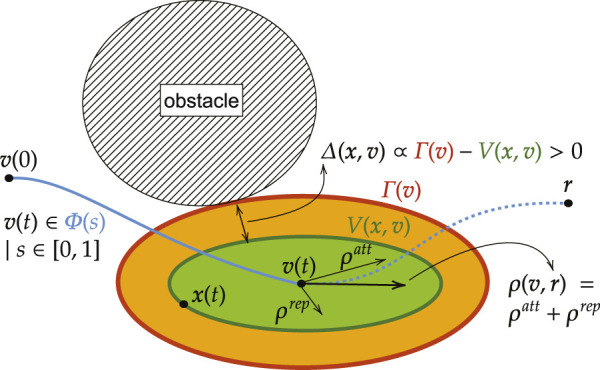
Basic Idea of the Invariant Level Set Explicit Reference Governor − The spherical obstacle is avoided by moving the applied reference v(t) over the a priori unknown (i.e. non pre-computed) path Φ(s) of steady-state admissible equilibria. The green ellipsoid represents the invariant level set value V(x,v) which embeds the future trajectory of x(t) if the current v(t) were to remain constant. The orange ellipsoid represents the threshold value Γ(v) of the invariant level set that touches the obstacle constraint. The Dynamic Safety Margin (DSM) Δ(x,v) is proportional to the difference between these level-set values and represents how safe it is to change v(t) in the direction of the Navigation Field (NF) ρ(v,r), with attraction toward the desired reference r and repulsion away from obstacles.

However, rather than pre-computing a suitable trajectory v(t), the ERG achieves these objectives by continuously manipulating the derivative of the applied reference as follows

$$\dot{v}=\rho \left(v,r\right)\Delta \left(x,v\right),$$ (20)

where ρ(v,r) is the **Navigation Field** (NF), i.e. a vector field that generates the desired steady-state admissible path Φ(s), and Δ(x,v) is the **Dynamic Safety Margin** (DSM), i.e. a scalar that quantifies the “distance” between the transient dynamics of the pre-stabilized system and the constraint boundaries if the current v(t) were to remain constant. The principle behind the ERG framework is illustrated in Figure 4.

This section extends the ERG framework to handle the case of multi-agent systems. The main challenge is given by the fact that the Distributed ERG (D-ERG) solution must ensure the satisfaction of multi-agent coordination constraints $g\left(x_{i},x_{k}\right)\geq 0$, such as the collision avoidance constraints (10). These constraints are not only dependent on agent’s *i* own dynamics, but also on the dynamics of agents *k* with k≠i. Hence, the original ERG framework, presented in (Nicotra and Garone, 2018, Theorem 1), would require a single, centralized ERG scheme to enforce the full set of constraints $c\left(x_{1:N_{a}},v_{1:N_{a}}\right)\geq 0$ on the aggregated states and references. Computing a single, non-conservative DSM would be challenging. Moreover, this scheme would inherently limit the velocity of the aggregate reference ${\dot{v}}_{1:N_{a}}$ based on the agent that is closest to constraint violation, resulting in poor performance.

**FIGURE 5:**
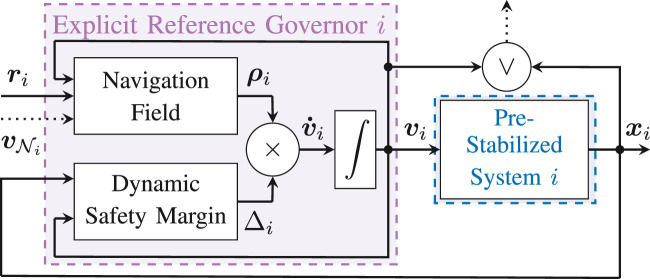
Distributed Explicit Reference Governor (D-ERG) Scheme for Agent *i*. To ensure that the constraints are satisfied for any desired reference configuration $r_{i}$, the ERG manipulates the rate of change of the applied reference ${\dot{v}}_{i}$ by computing a suitable Navigation Field (NF) $\rho_{i}$ and a Dynamic Safety Margin (DSM) ${\text{Δ}}_{i}$. The NF determines the current direction of ${\dot{v}}_{i}$ and the DSM regulates the modulus of ${\dot{v}}_{i}$ such that dynamic transients do not cause constraint violations. Agent *i* relies on the signal $v_{N_{i}}$, as defined in Figure 2, that is available in its local one-hop neighborhood and communicates its own signals $v_{i}$ or $x_{i}$ (but not both) with its neighboring agents, making the ERG distributed.

Here, the objective is to show that it is possible to ensure convergence and constraint satisfaction for the overall swarm by manipulating the reference of each agent in a distributed fashion as follows

$${\dot{v}}_{i}=\rho \left(v_{N_{i}},r_{i}\right)\text{Δ}\left(x_{i},v_{i}\right),$$ (21)

with $v_{N_{i}}$ defined in Figure 2. The proposed solution computes a DSM for each agent and is based on decomposing the multi-agent coordination constraints $g\left(x_{i},x_{k}\right)\geq 0$ into an auxiliary constraint on the references, i.e. $\gamma_{1}\left({\bar{x}}_{v_{i}},{\bar{x}}_{v_{k}}\right)\geq \delta$, and an auxiliary constraint on the dynamics of the individual agents, i.e. $\gamma_{2}\left(x_{i},v_{i}\right)\geq 0$, which can be accounted for in the NF and the DSM, respectively. In what follows $h\left(x_{i},v_{i}\right)\geq 0$ denotes the set of agent independent constraints, such as constraints (7)–(9). The rest of this section provides the updated definitions of the NF $\rho \left(v_{N_{i}},r_{i}\right)$ and the DSM $\Delta \left(x_{i},v_{i}\right)$ used in (21) by identifying sufficient conditions for the correct behavior of the D-ERG, as proven in Theorem 1. The schematic representation of the D-ERG is illustrated in Figure 5.

DEFINITION 1 (Navigation Field). Let the NF $\rho \left(v_{N_{i}},r_{i}\right)$ be such that, for any possibly time-varying piecewise continuous reference $r_{1:N}$, the initial value problem

$$\{\begin{array}{l} {\dot{\nu }}_{i}\left(\tau \right)=\rho \left(\nu_{N_{i}}\left(\tau \right),r_{i}\right),\\ \nu_{i}\left(0\right)=v_{i}, \end{array}$$ (22)

satisfies the following.

1. $\left|\left|\rho \left(\nu_{N_{i}},r_{i}\right)\right|\right|$ is finite for all possible $\left(\nu_{N_{i}},r_{i}\right)$;
2. $h\left({\bar{x}}_{v_{i}},v_{i}\right)\geq \delta \Rightarrow h\left({\bar{x}}_{v_{i}\left(\tau \right)},\nu_{i}\left(\tau \right)\right)\geq \delta ,\forall \tau \geq 0$ *;*
3. $\gamma_{1}\left({\bar{x}}_{v_{i}},{\bar{x}}_{v_{k}}\right)\geq \delta \Rightarrow \gamma_{1}\left({\bar{x}}_{v_{i}\left(\tau \right)},{\bar{x}}_{v_{k}\left(\tau \right)}\right)\geq \delta ,\forall \tau \geq 0$;
4. For any constant reference $r_{1:N}$, there exists a non-empty set of initial conditions V such that $\forall v_{1:N}\in V$, then

$$h\left({\bar{x}}_{r_{1:N}},r_{1:N}\right)\geq \delta \wedge \gamma_{1}\left({\bar{x}}_{r_{1:N}}\right)\geq \delta \Rightarrow \underset{\tau \to \infty }{\text{lim}}\nu_{1:N}\left(\tau \right)=r_{1:N}.$$

The key takeaway from Definition 1 is that it only considers the first-order dynamics (22). Thus, the NF is only responsible for generating a steady-state admissible path that connects the current references $v_{1:N}$ to the target references $r_{1:N}$. Since the NF does not account for the system dynamics, we refer to δ>0 as the “static safety margin”.

DEFINITION 2 (Dynamic Safety Margin). Let the DSM $\Delta \left(x_{i},v_{i}\right)$ be such that the solution of the initial value problem

$$\{\begin{array}{l} {\dot{\xi }}_{i}\left(\tau \right)=f\left(\xi_{i}\left(\tau \right),v_{i}\right),\\ \xi_{i}\left(0\right)=x_{i}, \end{array}$$ (23)

satisfies the following.

1. $\Delta \left(x_{i},v_{i}\right)>0\Rightarrow h\left(\xi_{i}\left(\tau \right),v_{i}\right)>0,\forall \tau \geq 0$;
2. $\Delta \left(x_{i},v_{i}\right)>0\Rightarrow \gamma_{2}\left(\xi_{i}\left(\tau \right),v_{i}\right)>0,\forall \tau \geq 0$;
3. $\Delta \left(x_{i},v_{i}\right)\geq 0\Rightarrow h\left(\xi_{i}\left(\tau \right),v_{i}\right)\geq 0,\forall \tau \geq 0$;
4. $\Delta \left(x_{i},v_{i}\right)\geq 0\Rightarrow \gamma_{2}\left(\xi_{i}\left(\tau \right),v_{i}\right)\geq 0,\forall \tau \geq 0$;
5. $\Delta \left(x_{i},v_{i}\right)=0\Rightarrow \text{Δ}\left(\xi_{i}\left(\tau \right),v_{i}\right)\geq 0,\forall \tau \geq 0$;
6. ∀δ>0, ∃ε>0 such that

$$h\left({\bar{x}}_{v_{i}},v_{i}\right)\geq \delta \wedge \gamma_{1}\left({\bar{x}}_{v_{i}},{\bar{x}}_{v_{k\neq i}}\right)\geq \delta \Rightarrow \text{Δ}\left({\bar{x}}_{v_{i}},v_{i}\right)\geq \varepsilon .$$

The intuition behind the DSM is that it quantifies the distance between the constraints and the transient dynamics of the individual closed-loop system.

Theorem 1. Consider N identical pre-stabilized systems ${\dot{x}}_{i}=f\left(x_{i},v_{i}\right)$ such that, if the applied reference $v_{i}$ remains constant, the closed-loop equilibrium point ${\bar{x}}_{v_{i}}$ is asymptotically stable. Let each agent be subject to a set of agent-independent constraints $h\left(x_{i},v_{i}\right)\geq 0$ and a set of multi-agent coordination constraints $g\left(x_{i},x_{k}\right)\geq 0$ with i≠k. Moreover, let the auxiliary constraints $\gamma_{1}\left({\bar{x}}_{v_{i}},{\bar{x}}_{v_{k}}\right)\geq \delta$ and $\gamma_{2}\left(x_{i},v_{i}\right)\geq 0$ be defined so that

$$\begin{array}{cc} \gamma_{1}\left({\bar{x}}_{v_{i}},{\bar{x}}_{v_{k}}\right)\geq \delta & \wedge \gamma_{2}\left(x_{i},v_{i}\right)\geq 0\\ \gamma_{1}\left({\bar{x}}_{v_{k}},{\bar{x}}_{v_{i}}\right)\geq \delta & \wedge \gamma_{2}\left(x_{k},v_{k}\right)\geq 0 \end{array}\}\Rightarrow g\left(x_{i},x_{k}\right)\geq 0.$$ (24)

Given the navigation field $\rho \left(v_{N_{i}},r_{i}\right)$ and the dynamic safety margin $\text{Δ}\left(x_{i},v_{i}\right)$, let the initial conditions $x_{1:N}\left(0\right)$, $v_{1:N}\left(0\right)$ be such that $\Delta \left(x_{1:N}\left(0\right),v_{1:N}\left(0\right)\right)\geq 0$. Then, the D-ERG formulation (21) ensures constraint satisfaction, i.e.

• $h\left(x_{1:N}\left(t\right),v_{1:N}\left(t\right)\right)\geq 0,\;\forall t\geq 0$;
• $g\left(x_{i}\left(t\right),x_{k}\left(t\right)\right)\geq 0,\;\forall t\geq 0,\forall i\in \left\{1:N\right\},\forall k\neq i$,

for any piecewise continuous reference $r_{1:N}\left(t\right)$.

Moreover, given a constant aggregate reference $r_{1:N}$ satisfying $h\left({\bar{x}}_{r_{i}},r_{i}\right)\geq \delta$ and $g\left({\bar{x}}_{r_{i}},{\bar{x}}_{r_{k}}\right)\geq \delta$, with i∈{1:N},k≠i, the D-ERG formulation (21) also ensures convergence, i.e.

• $\lim_{t\to \infty }x_{1:N}\left(t\right)={\bar{x}}_{r_{1:N}}$,

as long as $v_{1:N}\left(t\right)\in V,\;\forall t\geq 0$.

*Proof*: As detailed in the proof of (Nicotra and Garone, 2018), Theorem 1, it can be shown that (21) ensures $\Delta \left(x_{i}\left(t\right),v_{i}\left(t\right)\right)\geq 0,\;\forall t\geq 0$. As a result, it follows by definition of the DSM that $h\left(x_{1:N}\left(t\right),v_{1:N}\left(t\right)\right)\geq 0$ and $\gamma_{2}\left(x_{1:N}\left(t\right),v_{1:N}\left(t\right)\right)\geq 0$, ∀t≥0. Moreover, it follows by definition of the NF that, for any piecewise continuous and non-negative signal Δ(t), the solution to ${\dot{v}}_{i}=\Delta \left(t\right)\rho \left(v_{N_{i}},r_{i}\right)$ satisfies $\gamma_{1}\left({\bar{x}}_{v_{i}\left(t\right)},{\bar{x}}_{v_{k}\left(t\right)}\right)\geq \delta$, ∀t≥0, ∀i∈{1:N}, ∀k≠i. As a result it follows from (24) that $g\left(x_{i}\left(t\right),x_{k}\left(t\right)\right)\geq 0$, ∀t≥0, ∀i∈{1:N}, ∀k≠i. Finally, the convergence result $\lim_{t\to \infty }x_{1:N}\left(t\right)={\bar{x}}_{r_{1:N}}$ follows from the property $\Delta \left({\bar{x}}_{v_{1:N}},v_{1:N}\right)\geq \varepsilon$, as detailed in the proof of (Nicotra and Garone, 2018, Theorem 1).▪

**FIGURE 6:**
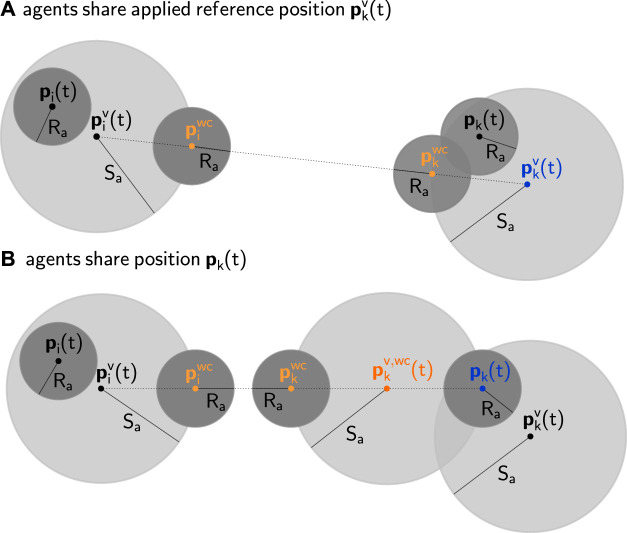
Geometric 2D representation of distributed collision avoidance between two pre-stabilized agents *i* **(left)** and *k* **(right)** with safety radii $R_{a}$ (dark gray disks), drawn from the perspective of agent *i*. The current position of each agent is $p_{i}\left(t\right)$, $p_{k}\left(t\right)$, whereas their current reference is $p_{i}^{v}\left(t\right),p_{k}^{v}\left(t\right)$. Due to the auxiliary constraint (38) (in light gray), accounted for in the DSM, the smallest possible distance between the two agents is equal to the distance between their worst-case future positions $p_{i}^{wc}$, $p_{k}^{wc}$. Together with the auxiliary constraint (37), which is enforced by the NF, this ensures the collision avoidance constraint (10). If the agents share their references (**case A**), agent *i* can compute the worst-case future position of agent *k* based on its current reference $p_{k}^{v}\left(t\right)$. If agent *i* only knows the position of agent *k* (**case B**), it must use $p_{k}\left(t\right)$ to compute the worst-case current reference $p_{k}^{v,wc}\left(t\right)$ and must then compute the worst-case future position based on $p_{k}^{v,wc}\left(t\right)$.

It is worth noting that, if V is equal to the entire set of steady-state admissible constraints, Theorem 1 implies convergence $\forall v_{1:N}\left(0\right)\in V$. However, if the NF admits deadlock configurations, the D-ERG will inherit the same limitations. The following subsections specialize the proposed D-ERG theory to the constrained control of a swarm of quadrotors. The choice of the auxiliary constraints that ensure multi-agent collision avoidance, as stated in (24), is illustrated in Figure 6. The pseudocode of the D-ERG is given in Algorithm 1, and the accompanying Table 1, which lists the type and amount of instructions to be executed, shows that the proposed D-ERG approach is computationally efficient and scalable.

**TABLE 1:** Computational Requirements of the D-ERG Algorithm − Type and amount of operations to be executed on-board an agent having in its one-hop local neighborhood $N_{\text{w}}$ walls, $N_{\text{o}}$ static obstacles, and $N_{\text{a}}-1$ dynamic agents. The required computations are basic arithmetic scalar, vector and matrix operations, scalar and vector min/max operations that scale approximately linear with the number of state constraints. Note that there is no iterative solver or matrix inversion required.

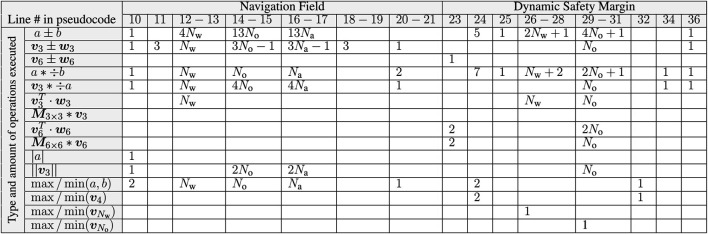

### 7.2 Navigation Field

As detailed in (Nicotra and Garone, 2018), the NF of agent *i* can be designed using a traditional attraction and repulsion field^1^

$$\rho \left(v_{N_{i}},r_{i}\right)=\rho_{i}^{\text{att}}+\rho_{i}^{\text{rep}},$$ (25)

Algorithm 1 : Pseudocode of the Distributed Explicit Reference Governor (D-ERG) Algorithm for Agent *i*.
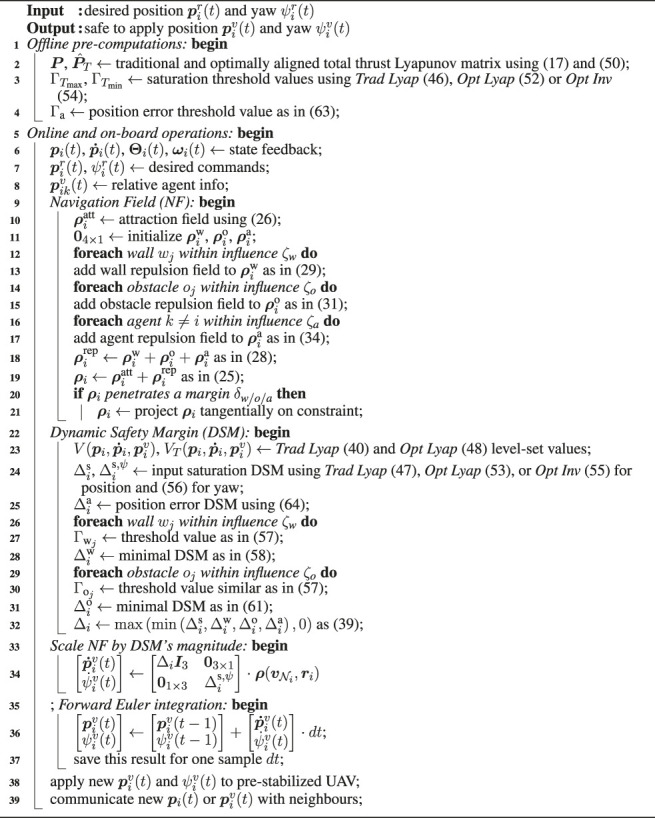

where the attraction field is

$$\rho_{i}^{\text{att}}={\left[l{\left(p_{i}^{r}-p_{i}^{v},\eta \right)}^{T},l\left(\psi_{i}^{r}-\psi_{i}^{v},\eta_{\psi }\right)\right]}^{T},$$ (26)

$\eta ,\eta_{\psi }>0$ are small smoothing radii chosen to avoid numerical problems when $\left\|r_{i}-v_{i}\right\|\to 0$, and

$$l\left(x,\eta \right)=\frac{x}{\max\left(\left\|x\right\|,\eta \right)}.$$ (27)

The repulsion field is the sum of linear repulsion fields pushing away from walls (w), obstacles (o), and nearby agents (a), i.e.

$$\rho_{i}^{\text{rep}}=\rho_{i}^{\text{w}}+\rho_{i}^{\text{o}}+\rho_{i}^{\text{a}}.$$ (28)

The repulsion field of all wall constraints is

$$\rho_{i}^{\text{w}}=-\sum_{j=1}^{N_{\text{w}}}\text{max}\left(\frac{\zeta_{\text{w}}-\left(d_{{\text{w}}_{j}}-R_{\text{a}}-c_{{\text{w}}_{j}}^{T}p_{i}^{v}\right)}{\zeta_{\text{w}}-\delta_{\text{w}}},0\right)\left[\begin{array}{c} {\hat{c}}_{{\text{w}}_{j}}\\ 0 \end{array}\right],$$ (29)

where $\zeta_{\text{w}}>0$ is the influence margin outside of which the repulsion field has no effect and $\delta_{\text{w}}\in \left(0,\zeta_{\text{w}}\right)$ is the static safety margin which guarantees that the reference is strictly steady-state admissible. The repulsion field of all static cylindrical obstacles includes the conservative (co) term

$$\rho_{i}^{\text{o},\text{co}}=-\sum_{j=1}^{N_{\text{o}}}\text{max}\left(\frac{\zeta_{{\text{o}}_{j}}-C_{j}\left(p_{i}^{v}\right)}{\zeta_{{\text{o}}_{j}}-\delta_{{\text{o}}_{j}}},0\right)\hat{\left[\begin{array}{c} {\left(o_{j}-p_{i}^{v}\right)}_{xy}\\ 0 \end{array}\right]},$$ (30)

with an influence margin $\zeta_{{\text{o}}_{j}}>0$, a static safety margin $\delta_{o_{j}}\in \left(0,\zeta_{o_{j}}\right)$ and $C_{j}\left(p_{i}^{v}\right)={\left\|p_{i}^{v}-o_{j}\right\|}_{xy}-\left(R_{o_{j}}+R_{\text{a}}\right)$. For spherical constraints, one can just use the full Euclidean norm and not project $\left(o_{j}-p_{i}^{v}\right)$ on the xy-plane. As detailed in (Koditschek and Rimon, 1990), however, conservative vector fields cannot achieve global stability in the presence of obstacle constraints. Therefore, the repulsion field also includes a non-conservative (n-co) term that destabilizes local saddle points

$$\rho_{i}^{\text{o}}=\rho_{i}^{\text{o},\text{co}}+\rho_{i}^{\text{o},\text{n}-\text{co}},$$ (31)

where

$$\rho_{i}^{\text{o},\text{ n}-\text{co}}=\{\begin{array}{ll} \alpha_{{\text{o}}_{j}}\sum_{j=1}^{N_{\text{o}}}\hat{\left[\begin{array}{c} o_{j}\left(2\right)-p_{i}^{v}\left(2\right)\\ -o_{j}\left(1\right)+p_{i}^{v}\left(1\right)\\ 0\\ 0 \end{array}\right]} & \text{if}\;\zeta_{{\text{o}}_{j}}\geq C_{j}\left(p_{i}^{v}\right),\\ & 0_{4\times 1}\text{if}\;\zeta_{{\text{o}}_{j}}<C_{j}\left(p_{i}^{v}\right) \end{array}$$ (32)

with circulation gain $\alpha_{{\text{o}}_{j}}>0$. For the case of a sphere, the term within brackets can be replaced by

$$\hat{\left[\begin{array}{c} -o_{j}\left(2\right)+p_{i}^{v}\left(2\right)+o_{j}\left(3\right)-p_{i}^{v}\left(3\right)\\ o_{j}\left(1\right)-p_{i}^{v}\left(1\right)-o_{j}\left(3\right)+p_{i}^{v}\left(3\right)\\ -o_{j}\left(1\right)+p_{i}^{v}\left(1\right)+o_{j}\left(2\right)-p_{i}^{v}\left(2\right)\\ 0 \end{array}\right]}.$$ (33)

In a similar way, one can define the repulsion field that acts on agent *i* caused by the other agents *k* as

$$\rho_{\text{i}}^{\text{a}}=\rho_{i}^{\text{a},\text{co}}+\rho_{i}^{\text{a},\text{n}-\text{co}},$$ (34)

where

$$\rho_{i}^{\text{a},\text{co}}=-\sum_{\begin{array}{c} k=1\\ k\neq i \end{array}}^{N_{\text{a}}}\max\left(\frac{\zeta_{\text{a}}-C_{ik}\left(p_{ik}^{v}\right)}{\zeta_{\text{a}}-\delta_{\text{a}}},0\right)\left[\begin{array}{c} {\hat{p}}_{ik}^{v}\\ 0 \end{array}\right],$$ (35)

with $C_{ik}\left(p_{ik}^{v}\right)={\left\|p_{ik}^{v}\right\|}_{xy}-2R_{\text{a}}-2S_{\text{a}}$, and

$$\rho_{i}^{\text{a},\text{n}-\text{co}}=\{\begin{array}{cc} \alpha_{\text{a}}\sum_{\begin{array}{c} k=1\\ k\neq i \end{array}}^{N_{\text{a}}}\hat{\left[\begin{array}{c} p_{ik}^{v}\left(2\right)\\ -p_{ik}^{v}\left(1\right)\\ 0\\ 0 \end{array}\right]} & \text{if}\;\zeta_{a}\geq C_{ik}\left(p_{ik}^{v}\right),\\ 0_{4\times 1} & \text{if}\;\zeta_{\text{a}}<C_{ik}\left(p_{ik}^{v}\right), \end{array}$$ (36)

with $\zeta_{\text{a}}>0$, $\delta_{\text{a}}\in \left(0,\zeta_{\text{a}}\right)$, $C_{ik}\left(p_{ik}^{v}\right)={\left\|p_{ik}^{v}\right\|}_{xy}-2R_{\text{a}}-2S_{\text{a}}$, and $\alpha_{\text{a}}>0$. This is sufficient to ensure the auxiliary constraint

$$\gamma_{1}\left(p_{ik}^{v}\right):{\left\|p_{ik}^{v}\right\|}_{xy}-2S_{a}-2R_{a}\geq \delta_{a}.$$ (37)

Following from Theorem 1, (24), agent collision can now be avoided by introducing the auxiliary constraint

$$\gamma_{2}\left(p_{i},p_{i}^{v}\right):S_{\text{a}}-\left\|p_{i}^{v}-p_{i}\right\|\geq 0.$$ (38)

As shown in Figure 6, the combination of (37) and (38) satisfies (10).

Remark 1. Equations (35) and (36) assume that agent i knows the difference between its own reference and the reference of agent k. However, the contribution of agent k becomes zero if ${\left|\left|p_{ik}^{v}\right|\right|}_{xy}\geq \zeta_{a}+2R_{a}+2S_{a}$. As a result, it is assumed that agents only share their reference with other agents within an inter-agent distance of $\zeta_{a}+2R_{a}+4S_{a}$. A possible option to eliminate communication entirely (i.e. a decentralized approach) is to have each agent measure the position of its neighbors (instead of communicating the applied references) and compute the worst-case references of the neighbors that would still ensure that (37) and (38) imply (10). This leads to two possible options

$$p_{ik}^{v}=\{\begin{array}{ll} p_{k}^{v}-p_{i}^{v} & i\;\mathrm{knows}\;p_{k}^{v}\\ p_{k}^{v,WC}-p_{i}^{v}=p_{k}-S_{a}\frac{p_{k}-p_{i}^{v}}{\left\|p_{k}-p_{i}^{v}\right\|}-p_{i}^{v} & i\;\mathrm{knows}\;p_{k}, \end{array}$$

where the latter has the advantage of not requiring inter-agent communication but also leads to a more conservative coordination strategy, as illustrated in Figure 6.

### 7.3 Dynamic Safety Margin

For each agent *i* its DSM, used in (21), can be obtained by taking the worst case DSM (i.e. the smallest one) of all active saturation (s), wall (w), obstacle (o), and agent collision (a) constraints^2^,

$$\Delta_{i}=\max\left(\text{min}\left({\text{Δ}}_{i}^{\text{s}},{\text{Δ}}_{i}^{\text{w}},{\text{Δ}}_{i}^{\text{o}},{\text{Δ}}_{i}^{\text{a}}\right),0\right)\geq 0.$$ (39)

For the offline design of the DSM we do not rely on explicit trajectory predictions, but use Lyapunov theory and optimization to design the DSM. As such, the following lemma is an important result used throughout this work to compute offline safe threshold values of Lyapunov level sets. As was visualized in Figure 4, it guarantees constraint satisfaction if the system dynamics never make its Lyapunov level set value V(x(t),v(t)) exceed that threshold value Γ(v(t)).

Lemma 2. Given a nonlinear pre-stabilized system $\dot{x}=f\left(x,v\right)$ with state vector x, applied reference v, equilibrium point ${\bar{x}}_{v}$, let

$$V\left(x,v\right)={\left(x-{\bar{x}}_{v}\right)}^{T}P\left(x-{\bar{x}}_{v}\right),\text{with}\;P>0,$$ (40)

be a Lyapunov function and let

$$c^{T}x\leq d\left(v\right)$$ (41)

be a linear constraint. Then, the Lyapunov treshold value

$$\Gamma \left(v\right)=\frac{{\left(-c^{T}{\bar{x}}_{v}+d\left(v\right)\right)}^{2}}{c^{T}P^{-1}c},$$ (42)

is such that V(x,v)≤Γ(v)⇒ (41).

*Proof*: See (Nicotra et al., 2019).▪

Since the DSM is computed on a per-agent basis, the agent index *i* will be omitted for the sake of notational simplicity. The following paragraphs address each constraint separately.

#### 7.3.1 Saturation Constraints

**FIGURE 7:**
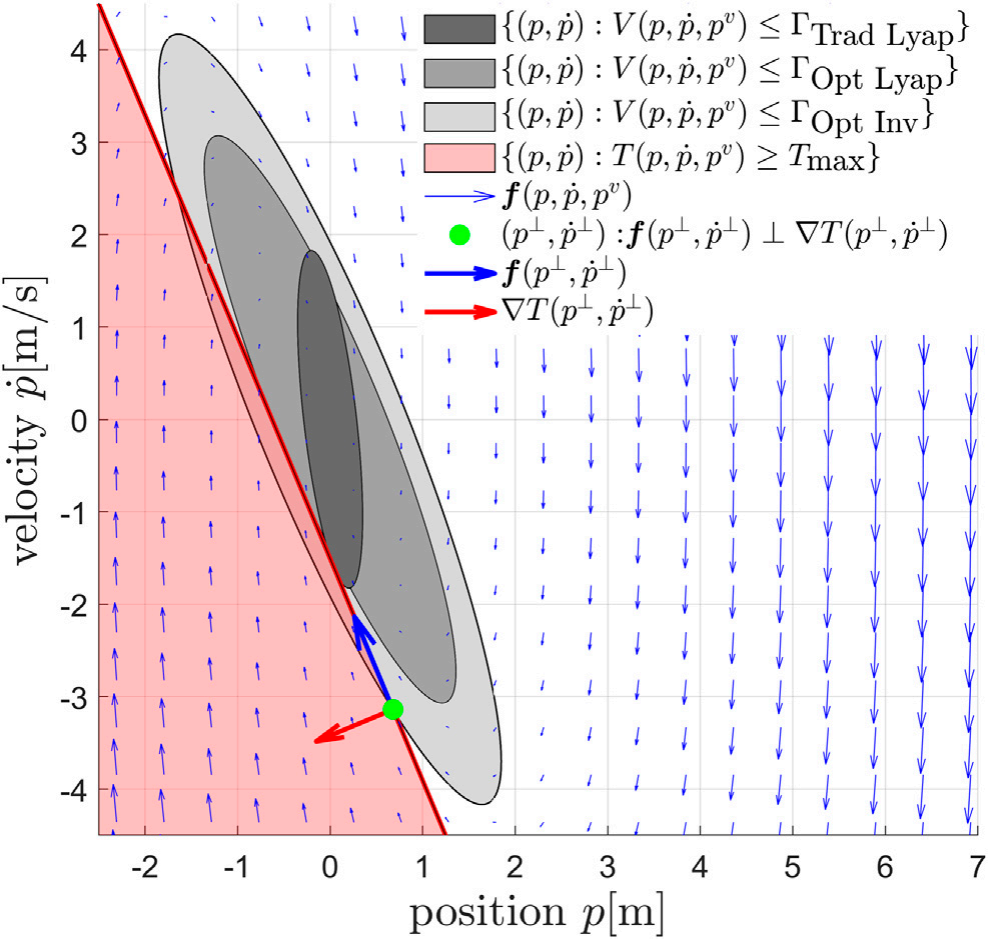
Phase plane representation of the proposed input constraint enforcement strategies, illustrated for a second-order dynamical system $m\ddot{p}=T-mg$ subject to the pre-stabilizing control law $T=m\left(k_{P}\left(p^{v}-p\right)-k_{D}\dot{p}+g\right)$ with $p^{v}=0$ and the input constraint $T\leq T_{\text{max}}$. The traditional Lyapunov based level-set (dark-grey) yields the most conservative DSM (47). Aligning the level-set with the constraints (medium gray) by solving the offline optimization problem (50) drastically increases the certified safe region (53). Further improvements can be obtained by solving (54) and using the invariant set which is the set obtained after subtracting the intersection between the light-grey Lyapunov level set and the region violating the input constraint from the light-grey Lyapunov level set. All three sets are certifiably safe since the flow vectors of the closed-loop system (in blue) all point inward. Note that, due to the high values in the first block diagonal of (17), any constraint that only depends on the position error variables, e.g. of the form p=a with a∈ℝ, is already very well aligned under the traditional strategy. Hence, performance benefits from optimal alignment are marginal.

In this section we show three strategies to compute a safe threshold value that ensure constraints on at least a subset of the inputs (5) are satisfied. The quantitative effects of these three strategies for an input constrained double integrator system are depicted in Figure 7.

*Traditional Lyapunov Level Set Strategy (Trad Lyap)*: One practical approach is to consider the outer loop control law and ensure the box constraints on the total thrust are satisfied,

$$T_{\min}\leq T=m\left\|K_{P}\left(p^{v}-p\right)-K_{D}\dot{p}+g{\hat{e}}_{3}\right\|\leq T_{\max}.$$ (43)

Since the inequality constraint (43) is nonlinear in the outer loop state variables, it is necessary to find a linear constraint that implies (43), in order to apply Lemma 2. A possible approach to provide a linear constraint is to make a distinction between the steady-state thrust $\mathrm{mg}{\hat{e}}_{3}$ and the dynamic feedback $m\left(K_{P}\left(p^{v}-p\right)-K_{D}\dot{p}\right)$. For the upper limit of the thrust constraint, this can be done by using the triangular inequality, and we obtain $T\leq m\left\|K_{P}\left(p^{v}-p\right)-K_{D}\dot{p}\right\|+\mathrm{mg}$. Hence,

$$\text{if}\;m\left\|K_{P}\left(p^{v}-p\right)-K_{D}\dot{p}\right\|+\mathrm{mg}\leq T_{\max}\Rightarrow T\leq T_{\max},$$ (44)

it is therefore sufficient to ensure that, $\forall e\in {\mathbb{R}}^{3}$:

$$\left[K_{P}{\hat{e}}^{T}-K_{D}{\hat{e}}^{T}\right]\left[\begin{array}{c} p^{v}-p\\ \dot{p} \end{array}\right]\leq \frac{T_{\max}-\mathrm{mg}}{m}.$$ (45)

This is equivalent to limiting the maximum acceleration of the UAV in any direction. The main interest with (45) is that it defines a rotationally invariant constraint that is linear for any given unitary vector $\hat{e}$, which can be expressed in the linear form (41) with $c={\left[c_{a}^{T},c_{b}^{T}\right]}^{T}$ by choosing $c_{a}=-K_{P}\hat{e}$, $c_{b}=-K_{D}\hat{e}$, and $d\left(p^{v}\right)=T_{\max}-\mathrm{mg}/m-K_{P}{\hat{e}}^{T}p^{v}$. Assuming unidirectional gains $K_{P}=k_{P}I_{3}$ and $K_{D}=k_{D}I_{3}$, the associated threshold value (42) is,

$$\Gamma_{T_{\max}}=\frac{1}{2}\frac{{\left(T_{\max}-\mathrm{mg}\right)}^{2}}{m^{2}}\frac{k_{P}+\varepsilon \left(1-\varepsilon \right)k_{D}^{2}}{k_{P}^{2}+k_{D}^{2}\left(k_{P}+\varepsilon k_{D}^{2}-2\varepsilon k_{P}\right)}.$$ (46)

Similarly, ${\text{Γ}}_{T_{\text{min}}}$ can be computed by replacing $T_{\text{max}}$ in (46) with $T_{\text{min}}$. The DSM that prevents the total thrust to saturate is

$$\Delta^{s}=\kappa_{s}\left(\min\left(\Gamma_{T_{\max}},\Gamma_{T_{\min}}\right)-V\left(p,\dot{p},p^{v}\right)\right),$$ (47)

with $\kappa_{s}\in {\mathbb{R}}_{>0}$.

*Optimally Aligned Lyapunov Level Set Strategy (Opt Lyap):* This section is an extension of the theory in (Garone et al., 2018) and applies it to higher-order quadrotor dynamics. Since linear systems are characterized by an infinite choice of quadratic Lyapunov functions, a way to improve the performance of the outer loop dynamics is to select the optimal Lyapunov based threshold value that is perfectly aligned with the total thrust constraints, instead of using (46), which is not aligned. Hence, one can find a common Lyapunov function in the quadratic form

$$V_{T}\left(p,\dot{p},p^{v}\right)={\left[\begin{array}{c} p-p^{v}\\ \dot{p} \end{array}\right]}^{T}P_{T}\left[\begin{array}{c} p-p^{v}\\ \dot{p} \end{array}\right],$$ (48)

with $P_{T}>0$ that satisfies the Lyapunov equation $A{\left(\tilde{R}\right)}^{T}P_{T}+P_{T}A\left(\tilde{R}\right)\leq 0$ and $A\left(\tilde{R}\right)$ defined in (6.2). By taking advantage of the rotational symmetry of the system and defining

$$P_{T}=\left[\begin{array}{cc} {\hat{P}}_{T,11}I_{3} & {\hat{P}}_{T,12}I_{3}\\ {\hat{P}}_{T,21}I_{3} & {\hat{P}}_{T,22}I_{3} \end{array}\right],$$ (49)

the optimal Lyapunov function can be obtained by solving the following linear matrix inequality

$$\{\begin{array}{l} \min\log\det\left({\hat{P}}_{T}\right)\;\text{subject to}:\\ A{\left(0\right)}^{T}{\hat{P}}_{T}+{\hat{P}}_{T}A\left(0\right)\leq 0\\ A{\left(\Delta \alpha \right)}^{T}{\hat{P}}_{T}+{\hat{P}}_{T}A\left(\text{Δ}\alpha \right)\leq 0\\ {\hat{P}}_{T}\geq c_{T}c_{T}^{T} \end{array},$$ (50)

where $\tilde{\alpha }$ and Δα are the current and the maximum allowed rotational error between ${\hat{z}}_{ℬ}$ and ${\hat{z}}_{ℬ}^{d}$, $c_{T}=-m{\left[k_{P},k_{D}\right]}^{T}$ and

$$A\left(\tilde{\alpha }\right)=\left[\begin{array}{cc} 0 & 1\\ -k_{P}\cos\left(\tilde{\alpha }\right) & -k_{D}\cos\left(\tilde{\alpha }\right) \end{array}\right].$$ (51)

Given the quadratic Lyapunov function (48), we obtain the threshold values

$$\begin{array}{l} \Gamma_{T_{\max}}=\frac{{\left(T_{\max}-\mathrm{mg}\right)}^{2}}{c_{T}^{T}{\hat{P}}_{T}^{-1}c_{T}},\\ \Gamma_{T_{\min}}=\frac{{\left(T_{\min}-\mathrm{mg}\right)}^{2}}{c_{T}^{T}{\hat{P}}_{T}^{-1}c_{T}}. \end{array}$$ (52)

The DSM that prevents the total thrust to saturate and is based on the Lyapunov function that is optimally aligned with this constraint, then becomes

$$\Delta^{s}=\kappa_{s}\left(\min\left(\Gamma_{T_{\max}},\Gamma_{T_{\min}}\right)-V_{T}\left(p,\dot{p},p^{v}\right)\right).$$ (53)

*Optimally Aligned Invariant Level Set Strategy (Opt Inv):* A more generic safe set can be obtained by considering the outer loop dynamics (19) with input (12) and computing offline the threshold value associated to the largest possible optimally aligned Lyapunov level set that satisfies the constraints of the following minimization problem

$$\left\{ \begin{array}{l} {\text{Γ}}_{T_{\text{max}/\text{min}}}=\underset{p,\dot{p},p^{v}}{\text{min}}V_{T}\left(p,\dot{p},p^{v}\right)\\ \text{subject}\;\text{to}:\\ \left\|T^{d}\left(p,\dot{p},p^{v}\right)\right\|=T\left(p,\dot{p},p^{v}\right)=T_{\text{max}/\text{min}}\\ f{\left(p,\dot{p},p^{v}\right)}^{T}\nabla T\left(p,\dot{p},p^{v}\right)\geq 0/\leq 0, \end{array}\right.$$ (54)

with the closed position loop dynamics $f\left(p,\dot{p},p^{v}\right)$ and the total thrust gradient $\nabla T\left(p,\dot{p},p^{v}\right)$. Doing so, one can obtain a safe invariant set by taking the optimally aligned Lyapunov level set and subtracting the inadmissible region, i.e. the region where the constraints are violated $T\geq T_{\max}$ or $T\leq T_{\min}$. The invariant set based DSM can be computed as,

$$\Delta^{\text{s}}=\kappa_{\text{s}}\min\left(\frac{\min\left(\Gamma_{T_{\max}},\Gamma_{T_{\min}}\right)-V_{T}\left(p,\dot{p},p^{v}\right)}{\left(\Gamma_{T_{\max}}+\Gamma_{T_{\min}}\right)/2},\underset{j\in \left\{1,2,3,4\right\}}{\min}\left(\frac{U_{\max}-U_{j}}{\left(U_{\max}-U_{\min}\right)/2},\frac{U_{j}-U_{\min}}{\left(U_{\max}-U_{\min}\right)/2}\right)\right).$$ (55)

Remark 2. To avoid motor saturation when tracking a non-zero yaw reference, it is also necessary to add an ERG on the yaw axis. This can be done using the NF in (26) and the DSM

$$\Delta^{s,\psi }=\kappa_{s,\psi }\underset{j\in \left\{1,2,3,4\right\}}{\min}\left(\frac{U_{\max}-U_{j}}{\left(U_{\max}-U_{\min}\right)/2},\frac{U_{j}-U_{\min}}{\left(U_{\max}-U_{\min}\right)/2}\right),$$ (56)

with $\kappa^{s,\psi }\in {\mathbb{R}}_{>0}$.

#### 7.3.2 Wall Constraints

The convex inequality constraints (8) are equivalent to (41) with $c={\left[c_{w_{j}}^{T},0_{3\times 1}^{T}\right]}^{T}$, and $d\left(p^{v}\right)=d_{w_{j}}-R_{a}$. As a result, the threshold value associated to the j-th wall constraint is

$$\Gamma_{w_{j}}=\frac{1}{2}\left(k_{P}+\varepsilon \left(1-\varepsilon \right)k_{D}^{2}\right){\left({\hat{c}}_{w}^{T}jp^{v}-d_{w_{j}}+R_{a}\right)}^{2}.$$ (57)

The dynamic safety margin corresponding to the wall constraint closest to violation then becomes,

$$\Delta^{w}=\kappa_{w}\left(\underset{j\in \left\{1,\ldots ,N_{w}\right\}}{\min}\left(\Gamma_{w_{j}}\right)-V\left(p,\dot{p},p^{v}\right)\right),$$ (58)

with $\kappa_{w}\in {\mathbb{R}}_{>0}$.

#### 7.3.3 Obstacle Constraints

Constraint (9) defines a non-convex admissible region. Given a fixed reference $p^{v}$, it can be shown using triangular inequalities that

$$\left\|p-o_{j}\right\|\geq \left\|p-p^{v}\right\|-\left\|p^{v}-o_{j}\right\|\geq R_{o_{j}}+R_{a}.$$ (59)

As a result, (9) can be enforced by simply ensuring

$${\hat{\left(p^{v}-o_{j}\right)}}^{T}\left(p^{v}-p\right)\geq R_{o_{j}}+R_{a}+\left\|p^{v}-o_{j}\right\|\geq 0.$$ (60)

The inequality constraints define a reference-dependent virtual wall and are equivalent to (41) with $c={\left[{\hat{\left(p^{v}-o_{j}\right)}}^{T},0_{3\times 1}^{T}\right]}^{T}$, and $d\left(p^{v}\right)={\hat{\left(p^{v}-o_{j}\right)}}^{T}p^{v}-R_{o_{j}}-R_{a}-\left\|p^{v}-o_{j}\right\|$. The DSM related to this constraint then becomes,

$$\Delta^{o}=\kappa_{o}\left(\underset{j\in \left\{1,\ldots ,N_{o}\right\}}{\min}\left(\Gamma_{o_{j}}\right)-V\left(p,\dot{p},p^{v}\right)\right).$$ (61)

with $\kappa_{\text{o}}\in {\mathbb{R}}_{>0}$.

#### 7.3.4 Agent Collision Avoidance

As explained in Section 7.2, collision avoidance can be satisfied by also enforcing the auxiliary constraint (38). Since constraint (38) applies equally in every direction in 3D space, it can be enforced using the Lyapunov threshold value associated to the linear constraint

$$\left[\begin{array}{cc} {\hat{e}}^{T} & 0_{3\times 1}^{T} \end{array}\right]\left[\begin{array}{c} p^{v}-p\\ \dot{p} \end{array}\right]\leq S_{a},\forall \hat{e}\in {\mathbb{R}}^{3}:\left\|\hat{e}\right\|=1,$$ (62)

thus leading to

$$\Gamma_{a}=\frac{1}{2}\left(k_{P}+\varepsilon \left(1-\varepsilon \right)k_{D}^{2}\right)S_{a}^{2}.$$ (63)

The DSM related to this constraint then becomes,

$$\Delta^{a}=\kappa_{a}\left(\Gamma^{a}-V\left(p,\dot{p},p^{v}\right)\right),$$ (64)

with $\kappa_{\text{a}}\in {\mathbb{R}}_{>0}$.

## 8 Results

We present the first results of an extensive experimental validation of the ERG and the D-ERG frameworks by means of single and multi-robot hardware experiments (a video of the experiments can be found at https://youtu.be/le6WSeyTXNU) using the experimental setup described hereafter. In a comparative simulation campaign we have analyzed statistically the goal and constraint satisfaction properties of our methodology. A summary of these results can be found in Section 9.

### 8.1 Experimental Setup

The experiments are performed using Crazyflie 2.1 nano-quadrotors in a Vicon motion capture system for indoor localization based on the Crazyswarm system architecture of (Preiss et al., 2017b). The computationally efficient control and navigation layers of Sections 6 and 7 are implemented in C and run at 500 Hz on-board the Crazyflie’s STM32F4 microprocessor’s firmware. The only programs running on the ground station are the special purpose motion capture tracker (Preiss et al., 2017b), a code for sending goal configurations to each quadrotor, and a code that mimics local communications between agents. Each UAV sends and receives new goal and feedback signals (i.e. the agent’s own state and neighbor information) via Crazyradios PA at 100 Hz. An on-board Kalman filter updates the agent’s own states at a higher rate than the motion capture system, but for the neighbor information such a Kalman filter update is not present. The experiment data is logged on-board the quadrotors on micro SD cards.

Each UAV is modeled with a static safety radius of $R_{\text{a}}=0.08\;\text{m}$ and a mass of approximately 34.6 g. Its inertia matrix $J=\text{diag}\left(17.31,17.94,33.75\right)\cdot 10^{-6}\;{\text{kgm}}^{2}$ is calculated from a CAD model and is only used to estimate the actuator torque constant. The estimated actuator thrust and torque constants amount $K_{T}=0.012\;\text{N}/{\text{V}}^{2}$ and $K_{\tau }=6.84\cdot 10^{-6}\;\text{Nm}/{\text{V}}^{2}$, respectively. The nominal distance between the motor axis and the center of mass of the aircraft amounts d=4.65 cm.

### 8.2 Tuning Guidelines

Here, we list guidelines for the tuning of the main parameters of the control and navigation layer and how this relates to the obtained performance and robustness. We advise users of this approach to tune the parameters in the order as they are listed below and to start with the input saturation constraints, followed by static and dynamic obstacle constraints.

1. First tune the inner loop gains ***K***_*R*_, ***K***_*ω*_ > 0 and then the outer loop gains ***K***_*P*_, ***K***_*D*_ > 0 for stable regulation control performance. The outer loop's settling time should be an order of magnitude slower than the one of the inner loop. This step is accomplished without worrying about the effect on any of the input or state constraints. The stiffer the pre-stabilized closed-loop system is tuned, the more the agents can be stacked in a smaller volume, at the cost of a more precise and higher rate odometry.
2. Eliminate numerical noise in the attraction field by selecting a strictly positive, but small value for the smoothing radius η.
3. Increase the DSM gains *κ* until no further performance increase is obtained. These gains are chosen such that the DSMs of the active constraints have the same order of magnitude.
4. Choose medium influence margins *ζ* defining from how far the obstacles are considered in the repulsion field. Too large values will require too large sensing ranges for static obstacles or communication ranges for dynamic obstacles, whereas too low values do not give enough reaction time.
5. For cooperative agent collision avoidance, choose the maximum position error radius S_a_. The larger this value, the higher the maximum attainable robot's speed, but the larger the distance traveled by each agent to reach its goal.
6. Select small circulation gains *α* around obstacles and agents to avoid robots getting stuck in local saddle points. Too large values tend to increase the settling time.
7. Choose strictly positive static safety margins *δ* to increase robustness. This also ensures the NF's repulsion term achieves its maximum amplitude while the DSM stays strictly positive. Hence this allows moving (and not blocking) the reference in directions pointing outward the obstacle constraint.

In all the experiments, the control gains of the inner-outer loop control law detailed in Section 6 are ***K***_*P*_ = 13.0 ***I***_3_, ***K***_*D*_ = 5.0 ***I***_3_, ***K***_*R*_ = diag(0.005, 0.005, 0.0003), and ***K***_*Ω*_ = diag(0.001, 0.001, 0.00005), which give moderately aggressive performance. The attraction field of the navigation layer is chosen with *η* = *η*_ψ_ = 0.005. Other parameters defined in Section 7 are specified in the following sections.

### 8.3 Single Aerial Robot Experiments

#### 8.3.1 Point-to-Point Transitions − Input Constraints

In the accompanying video we show that point-to-point transitions can easily destabilize a pre-stabilized quadrotor due to actuator saturation when the changes in $p^{v}$ become too abrupt.

The goal of the experiments is to validate the theory of Section 7.3.1 by showing that the navigation layer ensures safety for whatever $p^{r}$ and to quantify the difference in performance of the three strategies used to compute the DSM. To do so, we sequentially performed the following three experiments with a quadrotor where the navigation layer ensures input constraints satisfaction with $U_{\text{min}}=0.0\;\text{V}$, $U_{\text{max}}=3.5\;\text{V}$ or $T_{\text{min}}=0.0\;\text{N}$, $T_{\text{max}}=0.59\;\text{N}$ by using either:

- Trad Lyap: traditional Lyapunov-based DSM (47), with $\kappa_{\text{s}}=2.5$ and ε=0.5;
- Opt Lyap: optimally aligned Lyapunov-based DSM (53), with $\kappa_{\text{s}}=9.45$, Δα=0.349, ${\hat{P}}_{T,11}=0.8810$, ${\hat{P}}_{T,12}={\hat{P}}_{T,21}=0.3202$, ${\hat{P}}_{T,22}=0.1511$, $\Gamma_{T}=\min\left(\Gamma_{T_{\max}},\Gamma_{T_{\min}}\right)=0.266$;
- Opt Inv: invariance-based DSM (55), with $\kappa_{s}=1.80$, $\Gamma_{T}=3.00$.

**FIGURE 8:**
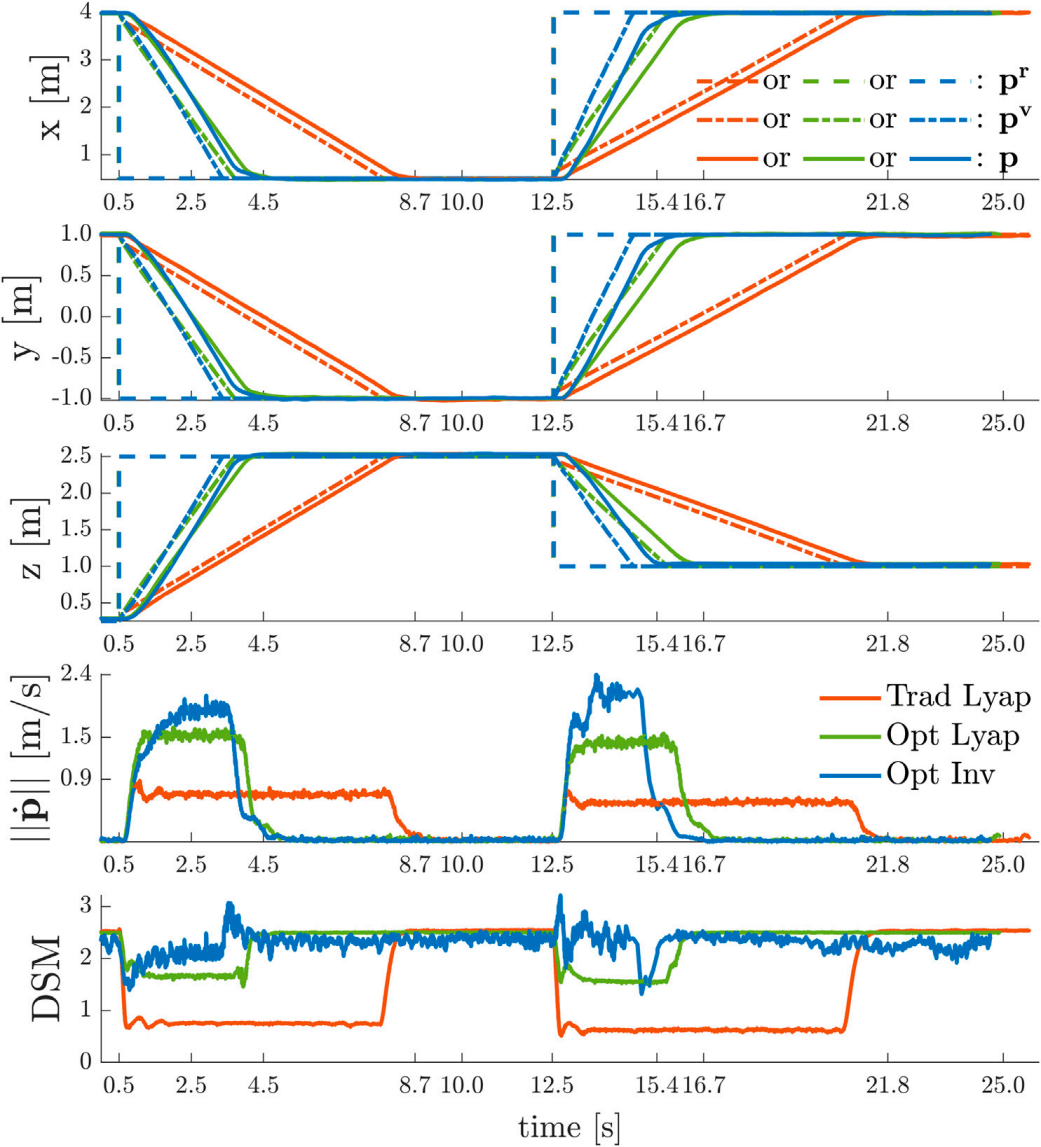
Point-to-Point Transitions Without Violation of Input Constraints − The three strategies for computing DSMs give provably stable and safe performance. The traditional Lyapunov strategy is the most conservative one, whereas the invariance based strategy outperforms the other two in terms of settling time and peak velocity.

As is depicted in Figure 8, in each of these experiments, the UAV starts from the initial hovering position $p\left(t\leq 0.5\;s\right)={\left[4.0,1.0,0.25\right]}^{T}\;\text{m}$, i.e. $\dot{p}\left(t\leq 0.5\;\text{s}\right)=0\;\text{m}/\text{s}$. At t=0.5 s and at t=12.5 s it is asked to transition between the points $p^{r}\left(0.5\;\text{s}\leq t<12.5\;\text{s}\right)={\left[0.50,-1.0,2.50\right]}^{T}\text{m}$ and $p^{r}\left(t\geq 12.5\;\text{s}\right)={\left[4.0,1.0,1.25\right]}^{T}\;\text{m}$.

The desired position set-point is always reached in a stable and safe (i.e. DSM≥0) manner. As expected from the theory in Section 7.3.1, a large reduction in settling time and an increase in the peak velocity is obtained when passing from a traditional Lyapunov based strategy, to the optimally aligned Lyapunov based strategy, and finally to the optimally aligned invariance based strategy. The latter gives the most aggressive performance and allows the aerial vehicle to obtain peak velocities of 2.4 m/s, which is about 2.76 times larger than what is obtained with the traditional Lyapunov based strategy. Note that the values of *κ* for these three cases where chosen such that the value of the DSMs are equal during hovering, i.e. when t∈[0.0,0.5] s, or t∈[8.7,12.5] s, or t∈[21.8,∞) s.

To show the effect of time-varying yaw angle references, we sequentially performed the following two experiments with the quadrotor using the invariance based ERG on the total thrust constraints and using either:

• no ERG on the yaw axis *ψ*;
• an ERG on yaw axis *ψ* as in (56) with $\kappa_{s,\psi }=1.80$.

**FIGURE 9:**
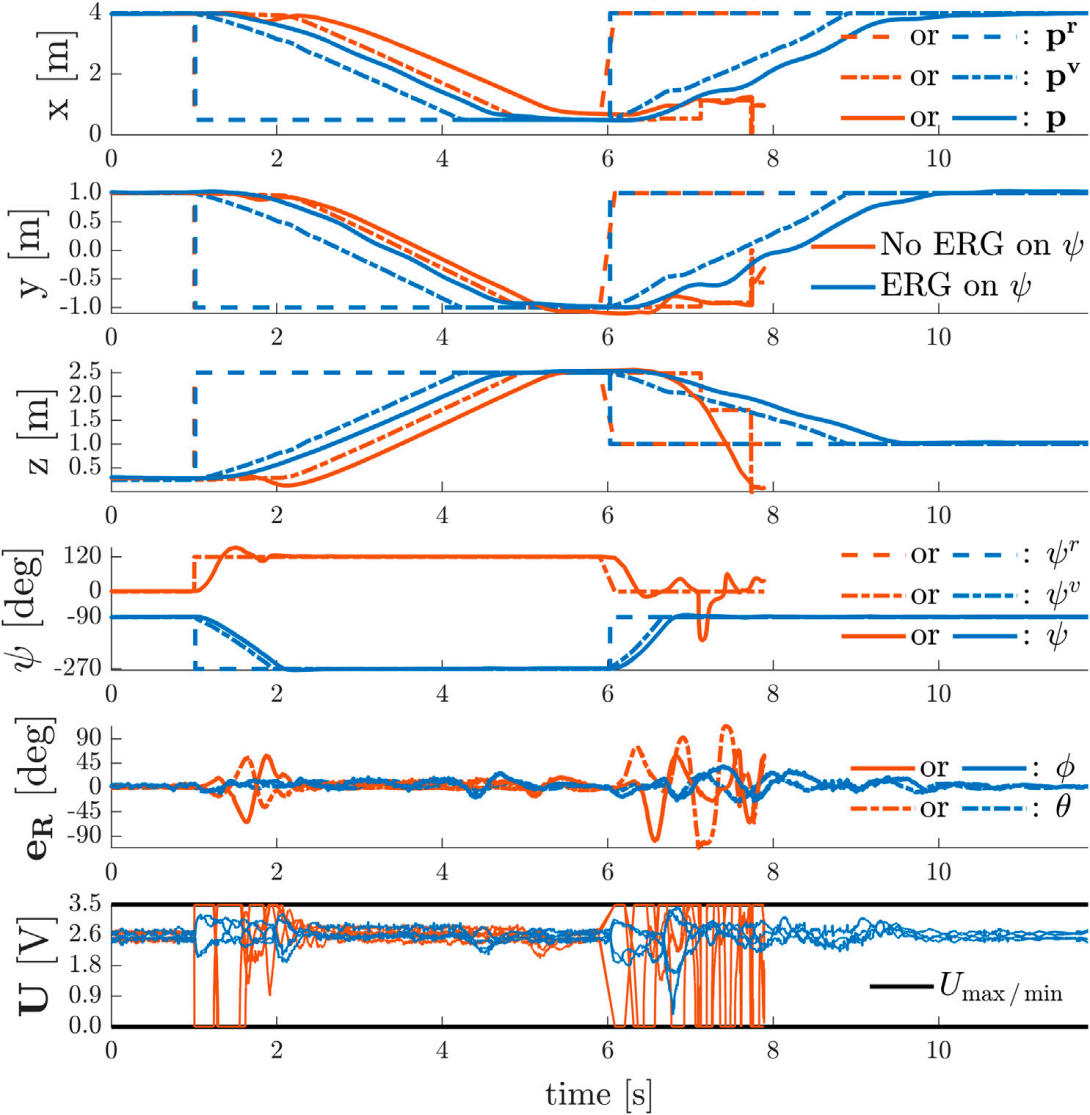
Simultaneous Point-to-Point Transitions and Discontinuous Yaw References With and Without Violation of Input Constraints − Besides an ERG on the position variables that limits the total thrust, an invariance based ERG on the yaw axis is required to ensure safety for non-stationary yaw references.

In each of these experiments, depicted in Figure 9, the UAV starts from the initial position $p\left(t\leq 1.0\;\text{s}\right)={\left[4.0,1.0,0.25\right]}^{T}\;\text{m}$ while hovering. At t=1.0 s and at t=6.0 s it receives the same position step references as in the previous experiment, but simultaneously it also receives yaw step references between $0^{\circ }$ and $120^{\circ }$ (No ERG on *ψ*), and between $-90^{\circ }$ and $270^{\circ }$ (ERG on *ψ*).

In the absence of an ERG on the yaw axis, the system remains stable under severe actuator saturation for the simultaneous position and yaw commands given at t=1.0 s but becomes unstable for the commands given at t=6.0 s. On the other hand, the system displays a stable, safe, and aggressive behavior during the whole experiment when the ERG is also applied to the yaw axis.

#### 8.3.2 Point-to-Point Transitions − Wall Avoidance

**FIGURE 10:**
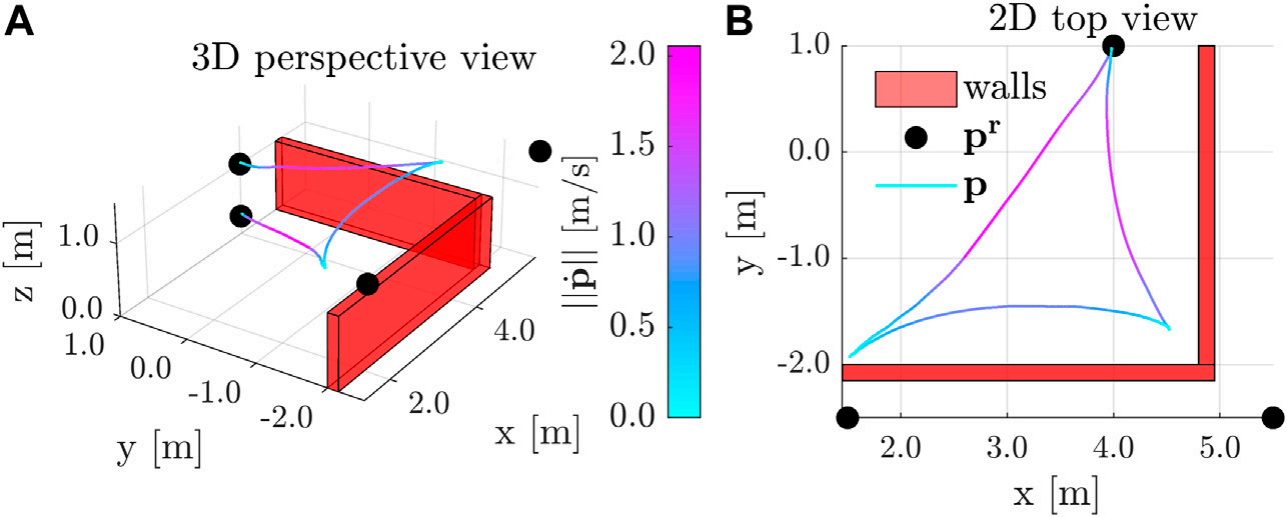
Point-to-Point Transitions with Wall Avoidance − The quadrotor achieves top speeds of 2 m/s and slows down as to avoid wall collisions, even when the position references are steady-state inadmissible.

The results depicted in Figure 10 show the aerial vehicle avoiding two virtual walls with $c_{w_{1}}={\left[1,0,0\right]}^{T}\;\text{m}$, $d_{w_{1}}=4.8\;\text{m}$, and $c_{{\text{w}}_{2}}={\left[0,-1,0\right]}^{T}\;\text{m}$, $d_{{\text{w}}_{2}}=2.0\;\text{m}$, when using an ERG with an invariance based DSM for the input constraints and a Lyapunov based DSM for the convex wall constraints with $\kappa_{w}=1.5$, $\zeta_{w}=1.0m$, and $\delta_{w}=0.01$ m. The UAV is initially hovering at ${\left[4.0,1.0,0.25\right]}^{T}\;\text{m}$ and is commanded consecutively to the positions ${\left[1.5,-2.5,1.5\right]}^{T}\;\text{m}$, ${\left[5.5,-2.5,1.5\right]}^{T}\;\text{m}$, and ${\left[4.0,1.0,1.0\right]}^{T}\;\text{m}$. From the logged data one can see that the quadrotor initially speeds up to a maximum speed of 2.0 m/s, and slows down such that overshoots do not cause collisions with the virtual walls. One can also see that the NF is designed such that it handles steady-state inadmissible references, which are depicted by black dots outside of the convex region in Figure 10.

### 8.4 Multiple Aerial Robots Experiments

In these experiments the UAVs are modeled as cylinders as detailed in Section 4.2.4, preventing them to fly over each other. Similarly to (Preiss et al., 2017a; Honig et al., 2018; Vukosavljev et al., 2019), this choice prevents a MAV’s propeller downwash effect to destabilize other MAVs which are flying closely underneath.

#### 8.4.1 Provably Safe Human-Swarm Teleoperation

**FIGURE 11:**
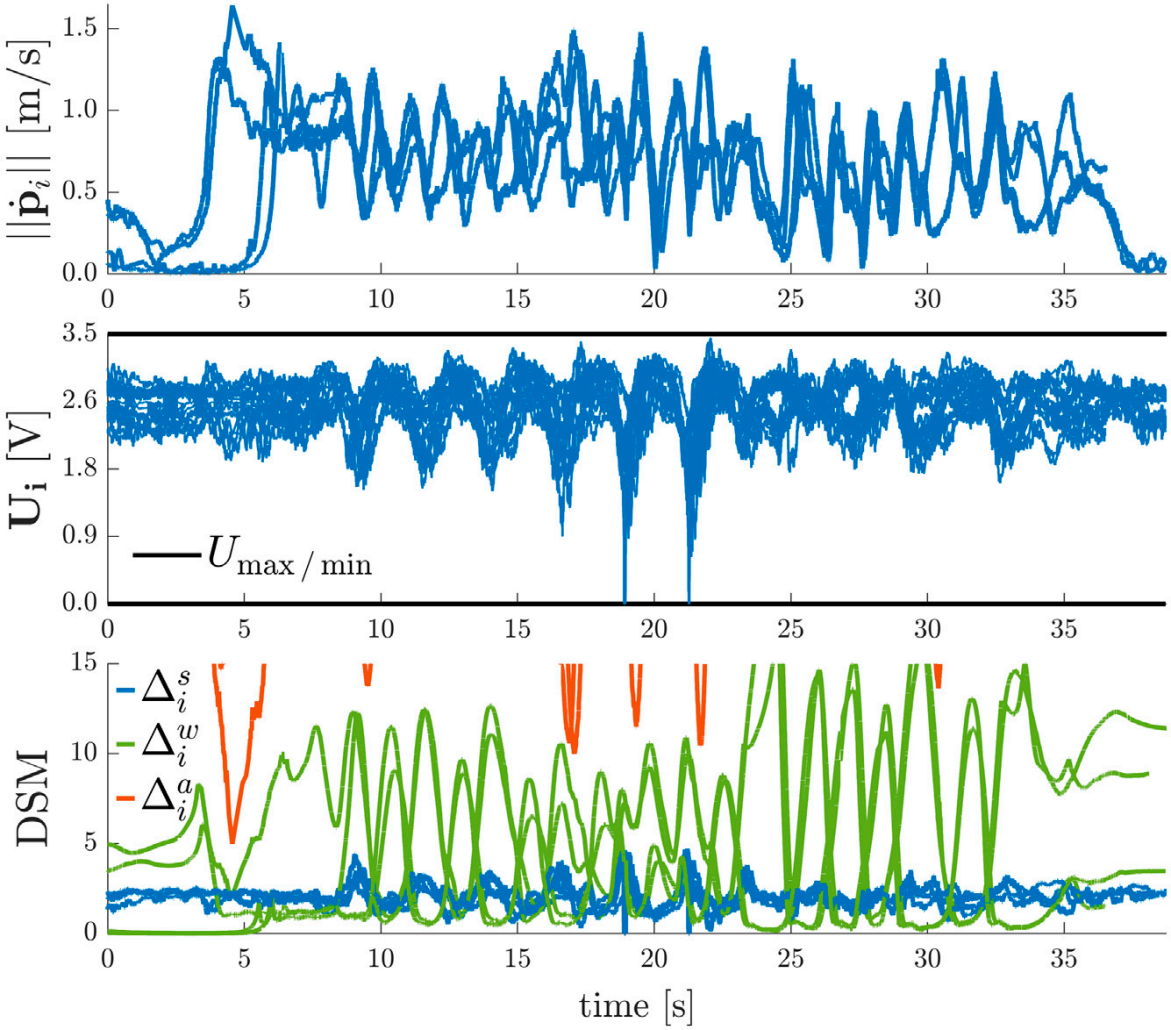
Results of the Human-Swarm Teleoperation Experiment in a Confined Environment − The D-ERG ensures the safe coordination of the quadrotor team formation. During the short periods where the actuator inputs come very close to their upper and lower limits (around t=20.0 s), the DSM decreases rapidly such that the applied reference is kept almost constant. Note that the steady-state motor voltages during hovering after t>37.0 s vary in a range of 2.1 V to 3.0  V. This is caused by variability in model parameters (e.g. battery displacements from the MAV's center of mass, different motor-propeller constants) and shows the robustness of the overall approach to model uncertainty.

In this experiment we show that the D-ERG ensures a swarm of $N_{a}=4$ quadrotors can be teleoperated by a human in a provably safe way within a confined environment composed of wall constraints with $c_{w_{1}}={\left[-1,0,0\right]}^{T}\;\text{m}$, $d_{{\text{w}}_{1}}=3\;\text{m}$, $c_{w_{2}}={\left[1,0,0\right]}^{T}\;\text{m}$, $d_{w_{2}}=4.8\;\text{m}$, $c_{w_{3}}={\left[0,-1,0\right]}^{T}\;\text{m}$, $d_{w_{3}}=2.0\;\text{m}$, and $c_{w_{4}}={\left[0,1,0\right]}^{T}\;\text{m}$, $d_{{\text{w}}_{4}}=1.5\;\text{m}$. We use the same ERG parameters as in Section 8.3.2, and for the collision avoidance between agents, we exchange $p^{v}$ between the agents and use $S_{a}=0.80\;\text{m}$, $\alpha_{a}=0.0$, $\zeta_{a}=1.50\;\text{m}$, $\delta_{a}=0.01\;\text{m}$, and $\kappa_{\text{a}}=50.0$. The human operator accelerates and decelerates the motion capture calibration wand fast in 3D space such as to exploit the quadrotor dynamics. Each agent is tasked to yaw in the direction of the wand and follows its relative position displacement. The logged data is depicted in Figure 11. The requested aggregated reference that wants to keep the swarm in a rigid square formation is deformed by the navigation layer by decreasing the rate of change of the reference applied to each pre-stabilized agent when it comes closer to violations of input, wall, or agent collision constraints. One can see that around t=20.0 s, there are short periods where the actuator inputs come very close to their upper and lower limits and ${\text{Δ}}_{i}^{\text{s}}$ is very close to zero, such that the applied reference is kept almost constant.

#### 8.4.2 Point-to-Point Transitions − Agent Collision Avoidance

**FIGURE 12:**
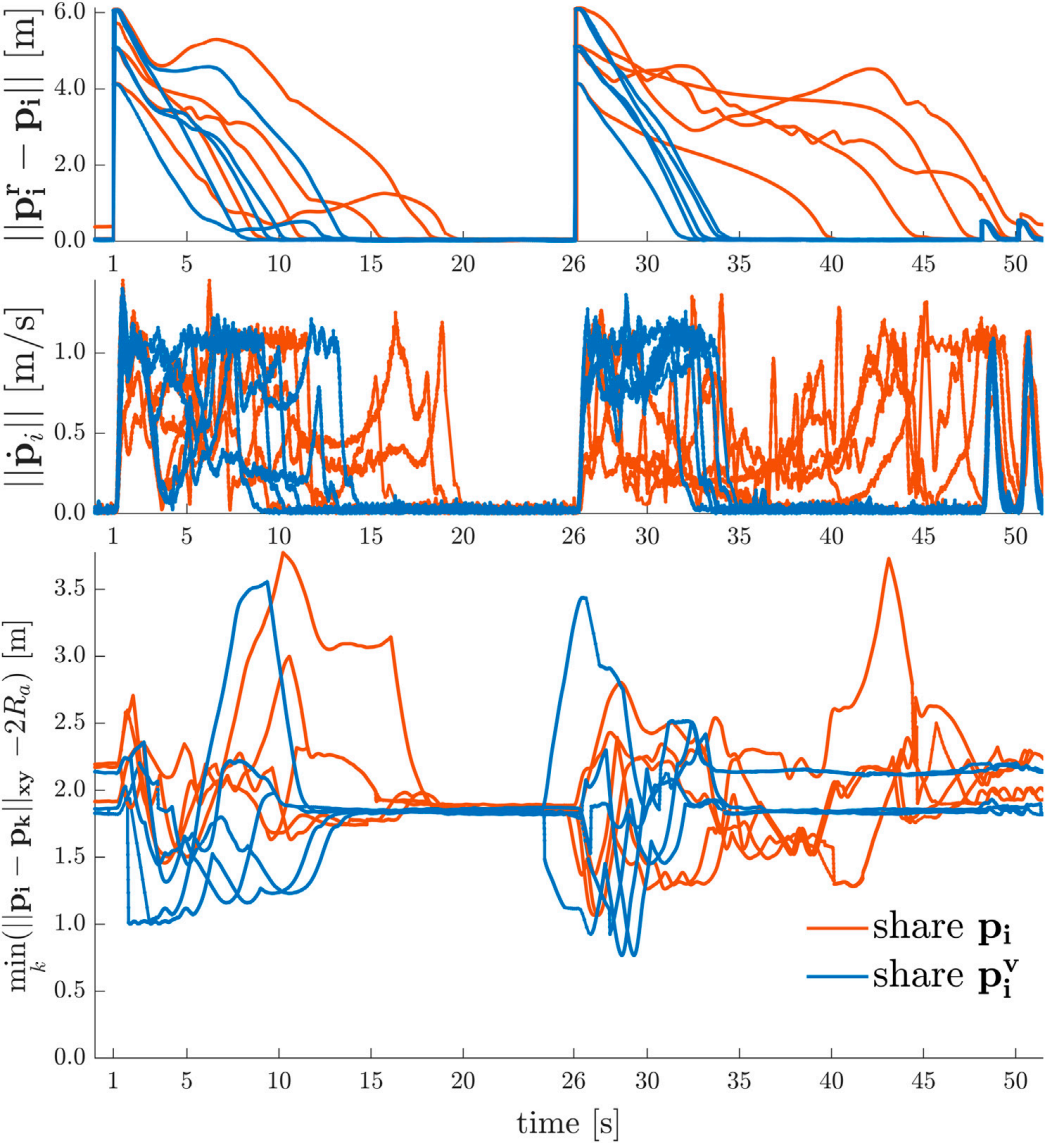
2D Point-to-Point Transitions with Agent Collision and Deadlock Avoidance − Exchanging the applied reference position $p_{i}^{v}\left(t\right)$ over the actual position $p_{i}\left(t\right)$ leads to a denser swarm, less distance traveled, and smaller settling times.

In Figure 12 the results of two experiments with a swarm of $N_{\text{a}}=5$ agents are depicted. Every agent is commanded to transition between specific goal positions at t=1.0 s and at t=26.0 s, such that if the agents are coordinated effectively, this globally leads to a line formation for the swarm. Moreover, they have to stay inside a confined environment bounded by four walls with $c_{w_{1}}={\left[-1,0,0\right]}^{T}\;\text{m}$, $d_{w_{1}}=4.8\;\text{m}$, $c_{w_{2}}={\left[1,0,0\right]}^{T}\;\text{m}$, $d_{{\text{w}}_{2}}=4.8\;\text{m}$, $c_{w_{3}}={\left[0,-1,0\right]}^{T}\;\text{m}$, $d_{{\text{w}}_{3}}=2.0\;\text{m}$, and $c_{w_{4}}={\left[0,1,0\right]}^{T}\;\text{m}$, $d_{{\text{w}}_{4}}=1.5\;\text{m}$. The navigation layer consists of a D-ERG using the parameters as in Section 8.4.1, but with $S_{\text{a}}=0.55\;\text{m}$, $\delta_{\text{a}}=0.1\;\text{m}$, $\alpha_{\text{a}}=0.1$. The same navigation task is performed twice, first by sharing p and then by sharing $p^{v}$ locally between the agents, as detailed in Remark 1. The results clearly show the D-ERG ensures every agent asymptotically reaches its desired position while avoiding collisions with other agents and the small circulation gain ensures the agents to not get stuck in local saddle points. Comparing the two cases one can see that sharing $p^{v}$ reduces the worst-case settling time over all agents for transitioning between formations by a factor of two. This is because the swarm remains more dense and the agents have to travel less distance. A potential drawback of the latter is that this explicitly requires communication between the agents, whereas sharing p could be communication-free (i.e. decentralized) if the agents would be equipped with sensors to measure inter-agent position vectors.

**FIGURE 13:**
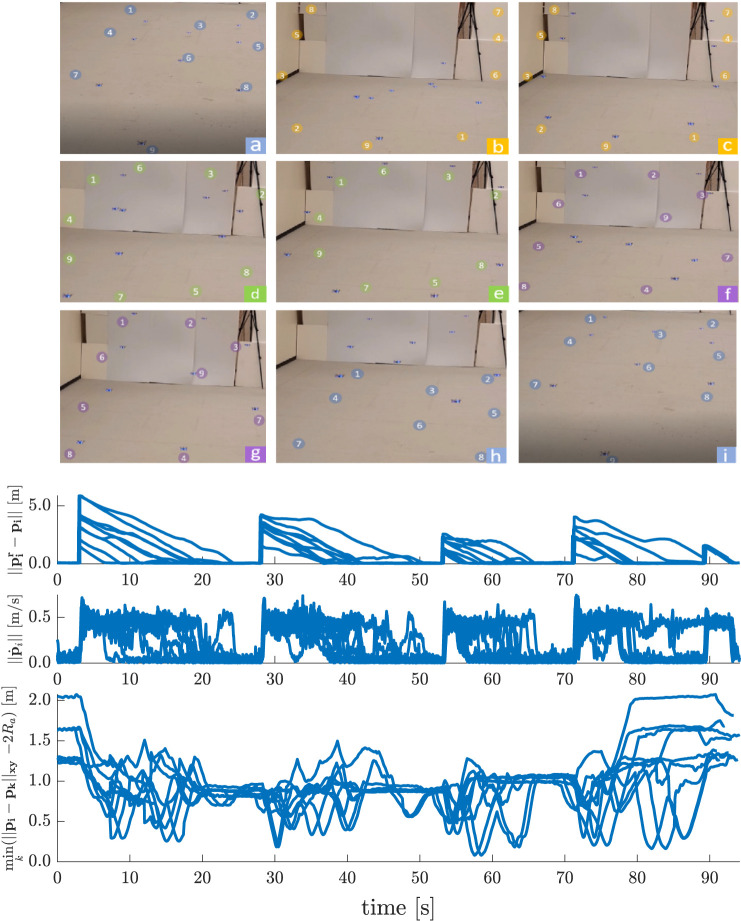
3D Point-to-Point Transitions with Agent Collision Avoidance − Asymptotically stable, collision free consecutive formations of the initials of the University of Colorado Boulder (UCB) are made. Nine consecutive shots **(a–i)** show the swarm members safely navigating from an initial configuration (in blue, shot a), to the U configuration (in yellow, shot c), to the C configuration (in green, shot e), to the B configuration (in purple, shot g), and finally back to the initial configuration (in blue, shot i).

Similar to the 2D line formation experiments, Figure 13 depicts the results of formation transitioning experiments in 3D with a swarm of $N_{\text{a}}=9$ agents. The actual applied reference positions $p_{i}^{v}$ between the agents are exchanged with $S_{\text{a}}=0.25\;\text{m}$, $\delta_{\text{a}}=0.01\;\text{m}$, and $\alpha_{\text{a}}=0.2$. Every agent is commanded to some set-point goal positions at t=3.0 s, at t=28.0 s, at t=53.0 s, at t=71.0 s, and at t=89.0 s that must be reached without causing any undesirable agent interactions such as collisions or deadlocks. Moreover the agents stay inside a confined environment bounded by the same four wall constraints. Note that since in this experiment $S_{\text{a}}$ is smaller than in Figure 12, this leads here to smaller peak velocities, but a more dense swarm (agents coming as close as 15 cm), since the agents have to travel less distance to avoid each other.

### 8.5 Analysis of Safety and Goal Satisfaction Certificates

In this simulation study we show some relevant statistics on the occurrence of constraint violations or deadlocks and compare the D-ERG with another optimization-free (i.e., closed form or explicit) approach solely based on attractive and repulsive Navigation Fields. The latter method is implemented by using the NF of Section 7.2 and by setting the DSM, which is a dynamic state-dependent and reference-dependent gain, to a user-tuned constant value. The latter can be interpreted as a fixed reference filter gain, which can only be selected before executing an experiment.

The results on safety and goal satisfaction for 3D point-to-point transitions of quadrotors in an increasingly densely filled environment with static obstacles and dynamic agents are depicted in Table 2. We use a cubic environment with side lengths of 16 m which is symmetrically centered in the origin. For each simulation we randomly place $N_{\text{o}}$ static spherical obstacles with $R_{\text{o}}=0.8\;\text{m}$, $\zeta_{\text{o}}=1\;\text{m}$, $\kappa_{\text{o}}=20$, and the initial and goal positions of $N_{\text{a}}$ quadrotors with $\zeta_{\text{a}}=1\;\text{m}$, $S_{\text{a}}=1.2\;\text{m}$, $\kappa_{\text{a}}=20$, $\kappa_{\text{s}}=6$, that exchange $p^{v}$ with their neighbors. This random placement is done under the condition that none of the influence margins are overlapping in steady-state. Hence, the swarm’s initial and desired position is at least steady-state admissible and convergence to the desired position of each agent can be detected as a static final error at the end of the simulation. For each defined combination of $N_{\text{o}}$ obstacles and $N_{\text{a}}$ agents, 500 random simulations are performed for each of the settings 1a), 1b), 2a), and 2b) depicted in Table 2. When there is at least one instability, one collision, or one deadlock detected in a simulation, the respective counters are incremented by one.

**TABLE 2:** Simulation Statistics on Safety and Goal Satisfaction − A cubic environment is randomly and increasingly densely filled with $N_{\text{o}}$ spherical obstacles and $N_{\text{a}}$ spherical agents doing 3D point to point transitions. The number of simulations that contain at least one instability, collision, or deadlock are denoted by #I, #C, and #D, respectively. Almost global asymptotic stability, with no collisions and no instabilities confirm the strong safety certificates of the D-ERG. This compared with another explicit approach solely based on Navigation Fields (NF).

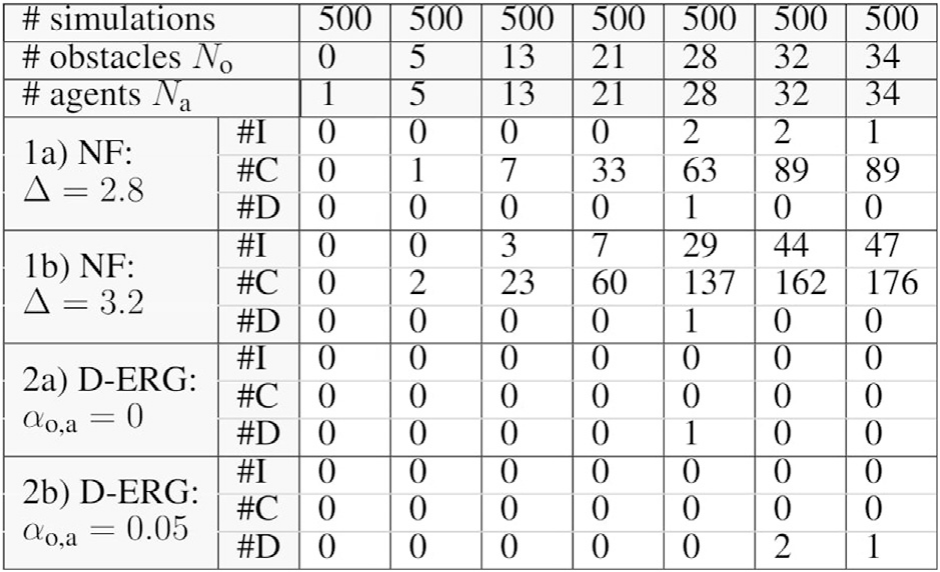

The strong safety certificates obtained when employing the D-ERG method are clear from the simulation data summarized in Table 2. The occurrence of instabilities and collisions is zero for the certified safe D-ERG, whereas for the Navigation Field (NF) method the occurrence is considerably large. When the constant reference gain in the NF approach is increased from Δ=2.8 to Δ=3.2, this leads to a larger number of collisions and instabilities due to severe control input saturation. For fair comparison, these DSM values were chosen around Δ=2.9, which is the steady-state value of the DSM in the D-ERG when a UAV hovers far away from obstacles.

For what concerns the goal satisfaction certificates, we observe almost global asymptotic stability. The statistical occurrence of deadlocks is almost negligible and only becomes measurable for very densely filled environments cluttered with agents and obstacles. Although a non-zero circulation gain ensures that pairs of agents cannot get stuck in local-saddle points, one can see that there is little benefit in using a circulation gain with a large number of agents. For some simulations it helps to avoid a deadlock, whereas in other simulations it can cause agents to get stuck in a local minimum. However, it is worth noting that this limitation is a consequence of the proposed NF and is not inherent to the D-ERG framework.

## 9 Discussion

In Section 8, we presented an extensive set of experimental and simulation studies of the proposed ERG and Distributed ERG framework, with the first real-world experiments to be found in the literature. These studies demonstrate the following key results (R) when applied to a homogeneous swarm of cooperative Crazyflie 2.1 quadrotors:

- R1: Computational efficiency allows high-rate real-time (500 Hz) computation of control commands on-board small UAVs with severely constrained CPU and RAM;
- R2: Almost globally asymptotically stable control performance for arbitrary position and yaw references (e.g. point-to point transitions or human-swarm teleoperation scenarios) for swarms in constrained environments. The measured statistical deadlock occurrence is negligible;
- R3: Provable safety under actuator inputs and state constraints, including collision avoidance between dynamical agents, and between agents and static obstacles;
- R4: Robustness in the presence of real-world uncertainties (e.g. non-modeled inner loop dynamics, variability of thrust and torque constants or battery voltages, battery displacement from center of mass, sensor noise, communication delays). The low-level control layer is proven to be robust to small attitude errors. Moreover, the D-ERG leverages the robustness of low-level controllers and maintains this property. Since the D-ERG’s DSMs itself relies on level-sets (i.e. Lyapunov or invariant set-based) and not on explicit state and input trajectory predictions to obtain safety guarantees, the overall approach is less model dependent and hence more robust;
- R5: Planner or reference agnostic safety certification with the ability to handle steady-state inadmissible references;
- R6: Offline ERG design strategies for the selection of safe threshold values to Lyapunov level-sets can lead to significant improvements in the control performance over traditional methods. Especially when the level sets are aligned with the constraints or when the more generic invariant safe sets are used with negligible increase of the on-board computational requirements.
- R7: The local nature of the D-ERG makes the algorithm scale very well with the number of agents. The distributed formulation that relies on local inter-agent distance and direction in applied reference positions (i.e. requiring agent communication) can lead to significantly smaller settling times and a denser swarm when compared to the decentralized formulation relying on inter-agent distance and direction in positions (i.e. requiring communication or exteroceptive sensing).

Algorithm 1 In future work, the proposed model-based add-on scheme can be further extended and combined with other control approaches, such as the adaptive control laws to deal with e.g. unmodeled dynamics, actuator deadzones as in (Wang et al., 2019; Yang et al., 2021a), and unavailable velocity measurements as in (Yang et al., 2021b) due to noisy low-cost sensors.

## 10 Conclusion

In this article we formulated the theory of a provably safe distributed constrained control framework, i.e., the Distributed Explicit Reference Governor (D-ERG), and demonstrated its efficacy on a homogeneous swarm of collaborative nano-quadrotors (i.e., a swarm of palm-sized Crazyflies 2.1) through multiple hardware and simulation experiments.

This approach has the following merits. Safety is guaranteed for agents with higher-order dynamics and with a large set of hard constraints such as the four actuator input limits and static and dynamic collision avoidance constraints. In contrast to optimization-based control schemes, this algorithm has a low cost of computation and memory and runs in real-time at a 500 Hz rate on-board the limited available robot hardware. Thereby, its local and reactive nature provides a good scalability to a large number of robots and obstacles. Since this add-on scheme only requires a pre-stabilized plant, it can be of great practical use when the controller is not accessible or not allowed to be changed, which is very often the case for commercial UAV flight control units. Its simple yet effective design makes it an interesting method for industrial robotic applications requiring safe real-time control systems.

However, some limitations still exist and can be addressed in future work. Since the Dynamic Safety Margin uses a single scalar to change the amplitude of the applied reference signal in the direction of the Navigation Field, the performance would reduce when applying this technique to systems with an increased state space dimension. Also, this robust level-set based D-ERG approach comes at the cost of an increased level of conservatism compared to approaches where the future trajectory is explicitly predicted or optimized for. Although the statistical occurrence of deadlocks is very low, the employed Navigation Field does not formally guarantee the absence of deadlocks.

## Data Availability

The raw data supporting the conclusion of this article will be made available by the authors, without undue reservation.

## Footnotes

1 dependency of ρ on $\left(v_{N_{i}},r_{i}\right)$ is omitted for simplicity of notation.

2 dependency of Δ on $\left(x_{i},v_{i}\right)$ is omitted for simplicity of notation.
